# Tumor microenvironment-activated single-atom platinum nanozyme with H_2_O_2_ self-supplement and O_2_-evolving for tumor-specific cascade catalysis chemodynamic and chemoradiotherapy

**DOI:** 10.7150/thno.73039

**Published:** 2022-07-04

**Authors:** Qiqi Xu, Yuetong Zhang, Zulu Yang, Guohui Jiang, Mingzhu Lv, Huan Wang, Chenghui Liu, Jiani Xie, Chengyan Wang, Kun Guo, Zhanjun Gu, Yuan Yong

**Affiliations:** 1 Key Laboratory of Pollution Control Chemistry and Environmental Functional Materials for Qinghai-Tibet Plateau of the National Ethnic Affairs Commission, School of Chemistry and Environment, Southwest Minzu University, Chengdu 610041, China.; 2 Key Laboratory of General Chemistry of the National Ethnic Affairs Commission, School of Chemistry and Environment, Southwest Minzu University, Chengdu 610041, China.; 3 CAS Key Laboratory for Biomedical Effects of Nanomaterials and Nanosafety, Institute of High Energy Physics, Chinese Academy of Sciences, Beijing100040, China.; 4 College of Pharmacy and Biological Engineering, Chengdu University, Chengdu, 610106, China.; 5 College of Pharmacy, Southwest Minzu University, Chengdu 610041, China.; 6 Department of Neurology, Affiliated Hospital of North Sichuan Medical College, Nanchong 637000, China.

**Keywords:** Single-atom nanozyme, tumor microenvironment-activated, enzyme-like activity, tumor catalytic therapy, chemodynamic and chemoradiotherapy

## Abstract

Nanozyme-based tumor collaborative catalytic therapy has attracted a great deal of attention in recent years. However, their cooperative outcome remains a great challenge due to the unique characteristics of tumor microenvironment (TME), such as insufficient endogenous hydrogen peroxide (H_2_O_2_) level, hypoxia, and overexpressed intracellular glutathione (GSH).

**Methods:** Herein, a TME-activated atomic-level engineered PtN_4_C single-atom nanozyme (PtN_4_C-SAzyme) is fabricated to induce the “butterfly effect” of reactive oxygen species (ROS) through facilitating intracellular H_2_O_2_ cycle accumulation and GSH deprivation as well as X-ray deposition for ROS-involving CDT and O_2_-dependent chemoradiotherapy.

**Results:** In the paradigm, the SAzyme could boost substantial ∙OH generation by their admirable peroxidase-like activity as well as X-ray deposition capacity. Simultaneously, O_2_ self-sufficiency, GSH elimination and elevated Pt^2+^ release can be achieved through the self-cyclic valence alteration of Pt (IV) and Pt (II) for alleviating tumor hypoxia, overwhelming the anti-oxidation defense effect and overcoming drug-resistance. More importantly, the PtN_4_C-SAzyme could also convert O_2_^·-^ into H_2_O_2_ by their superior superoxide dismutase-like activity and achieve the sustainable replenishment of endogenous H_2_O_2_, and H_2_O_2_ can further react with the PtN_4_C-SAzyme for realizing the cyclic accumulation of ∙OH and O_2_ at tumor site, thereby generating a “key” to unlock the multi enzymes-like properties of SAzymes for tumor-specific self-reinforcing CDT and chemoradiotherapy.

**Conclusions:** This work not only provides a promising TME-activated SAzyme-based paradigm with H_2_O_2_ self-supplement and O_2_-evolving capacity for intensive CDT and chemoradiotherapy but also opens new horizons for the construction and tumor catalytic therapy of other SAzymes.

## Introduction

Chemodynamic therapy (CDT), which takes advantage of iron-mediated Fenton reaction or Cu^2+^-/Mn^2+^-mediated Fenton-like reaction to convert less reactive H_2_O_2_ into highly toxic ·OH for inducing cell apoptosis and necrosis 1-4, has attracted widespread attention in recent years due to its high selectivity and logicality as well as activation of endogenous stimuli 5-9. To date, despite tremendous efforts have been achieved in triggering tumor cells apoptosis by the endogenous H_2_O_2_-dependent Fenton reaction in CDT 10-14, their therapeutic effectiveness remains a great challenge due to the tumor microenvironment (TME) characterized by insufficient endogenous hydrogen peroxide (H_2_O_2_, 50-100×10^-6^ M) 6,15-17, overexpressed glutathione (GSH) 18-24 as well as hypoxia microenvironment 25-32. Therefore, to potentially enhance their antitumor efficiency, several H_2_O_2_-supplementing strategies have been applied to convey exogenous H_2_O_2_ or trigger endogenous H_2_O_2_ generation in the tumor site 16, 33-37. Unfortunately, these methods still suffer from the drawbacks of the premature release of H_2_O_2_ and tumor hypoxia as well as sophisticated genetic engineering. Therefore, how to explore straightforward and evolutionary paradigm capable of H_2_O_2_-supplementing *in situ*, overcoming tumor hypoxia and constantly depleting GSH is urgently required but challenging for enhanced CDT, thus greatly breaking through therapeutic resistance and eradicating the tumor completely.

As we all known, hypoxia, as another characteristic feature of TME, is a barrier to tumor therapy and responsible for tumor migration, invasion, metastasis, and resistance 38-41. On account of this, chemoradiotherapy is usually utilized as a combination therapy for attenuating tumor hypoxia and lessening the chance of tumor recurrence clinically 42-44. So far, the reprogramming method of tumor hypoxia in chemoradiotherapy includes not only internal unique pathological stimuli by utilizing some smart nanomedicines delivering O_2_-triggering agents 45-49, but also hypoxic cell sensitization by the introduction of high-Z radiosensitizer or chemotherapeutics 50-53. Nevertheless, these relevant strategies for overcoming hypoxia commonly involve poor therapeutic effect or serious side effects on normal tissue. Therefore, in order to overcome tumor hypoxia and endogenous H_2_O_2_ deficiency simultaneously, it is essential and highly desirable to develop a versatile and TME-initiated nanomedicine, which has with the capacity of irradiation deposition, substantial drug release, persistent O_2_ and H_2_O_2_ cyclic replenishment at tumor site, thereby greatly improving the efficiency of CDT and chemoradiotherapy for synergistically eradicate tumor.

Nanozymes, with intrinsic enzyme-like characteristics, have sparked increasing interest in the biomedical field due to their tunable catalytic activity, high stability, and low cost 17, 54-61. Among the various reported paradigms, single-atom nanozymes (SAzymes) as a special kind of nanozymes with maximum atomic utilization efficiency, high density active sites and superior multienzyme-like activity as well as selectivity has emerged as prospective platform for TME-activated nanozyme-based tumor-specific catalytic therapy 62-67. By utilizing these unique superiorities, herein, a novel and TME-activated PtN_4_C-SAzyme was proposed to achieve continuous H_2_O_2_ self-replenishment, O_2_-evolving and GSH elimination as well as Pt^2+^ release for intensive synergistic CDT and chemoradiotherapy. The resultant system possesses several attractive advantages: i) The SAzyme could boost substantial ∙OH and O_2_ generation by their admirable peroxidase- and catalase-like activity as well as X-ray deposition capacity, which facilitated to oxygenate TME for intensive CDT and O_2_-dependent chemoradiotherapy; ii) Meanwhile, the self-cyclic valence alteration of Pt (IV) and Pt (II) endowed continuous depletion of intracellular overexpressed GSH and substantial Pt^2+^ release to overwhelm the anti-oxidation defense effect for enhanced tumor-specific therapy, ultimately; iii) More importantly, the PtN_4_C-SAzyme could also convert O_2_^·-^ into H_2_O_2_ and achieve the sustainable replenishment of H_2_O_2_ *in situ* of tumor, and H_2_O_2_ can further react with the PtN_4_C-SAzyme for realizing the cyclic accumulation of ∙OH and O_2_, thereby in turn generating a “key” to unlock the multiple enzyme-like properties of SAzymes for tumor-specific CDT and chemoradiotherapy. Taken together, our work provides a new TME-activated SAzyme-based high-performance anti-tumor paradigm with the ability to self-supplement H_2_O_2_ and boost O_2_-evolving as well as deplete intracellular GSH for tumor-specific cascade catalytic CDT and chemoradiothearpy.

## Results and discussion

**Scheme 1:**
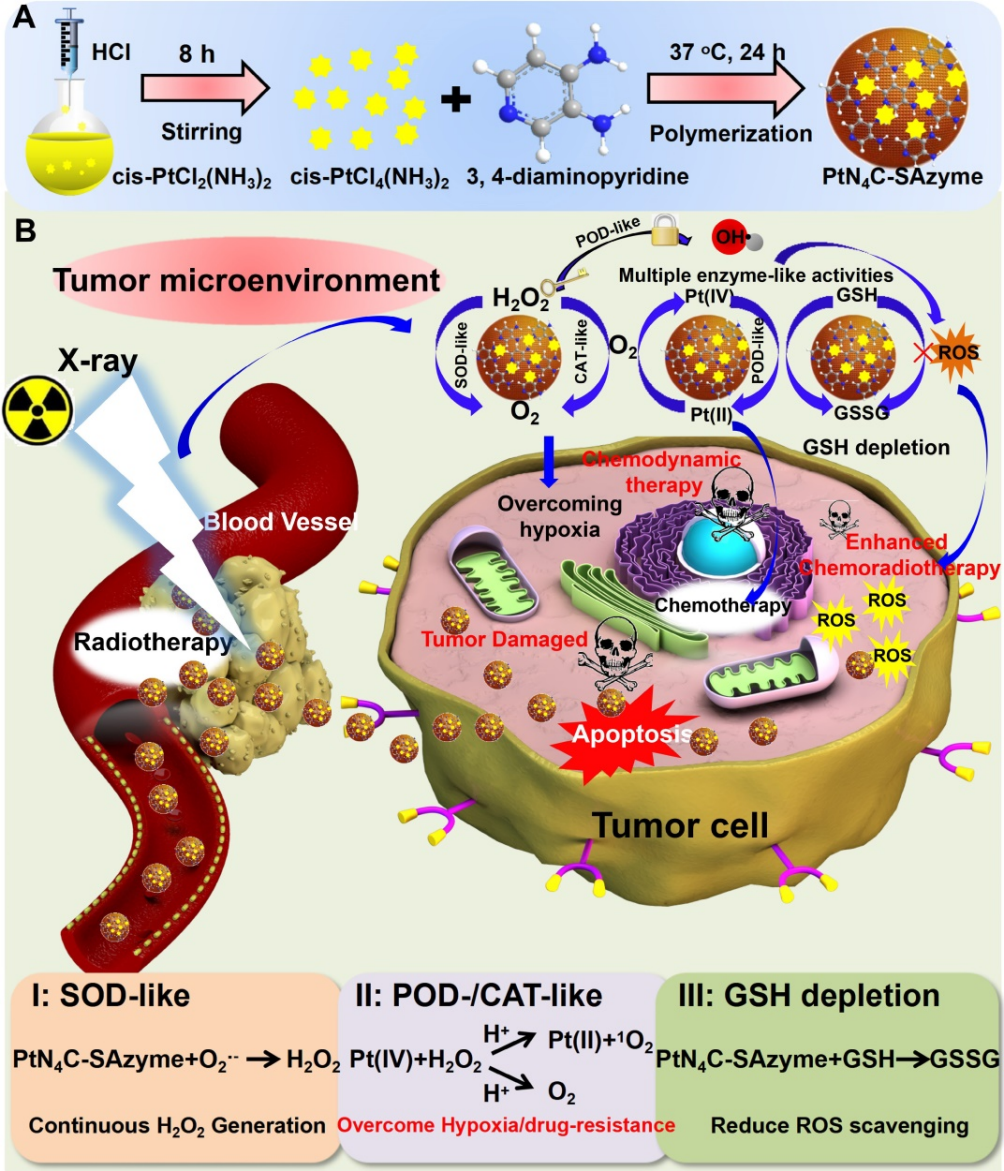
Schematic illustration of (A) the preparation process of PtN_4_C-SAzyme and (B) its cascaded catalytic reaction in tumor microenvironment for tumor synergistic enhanced CDT and chemoradiotherapy.

**Figure 1:**
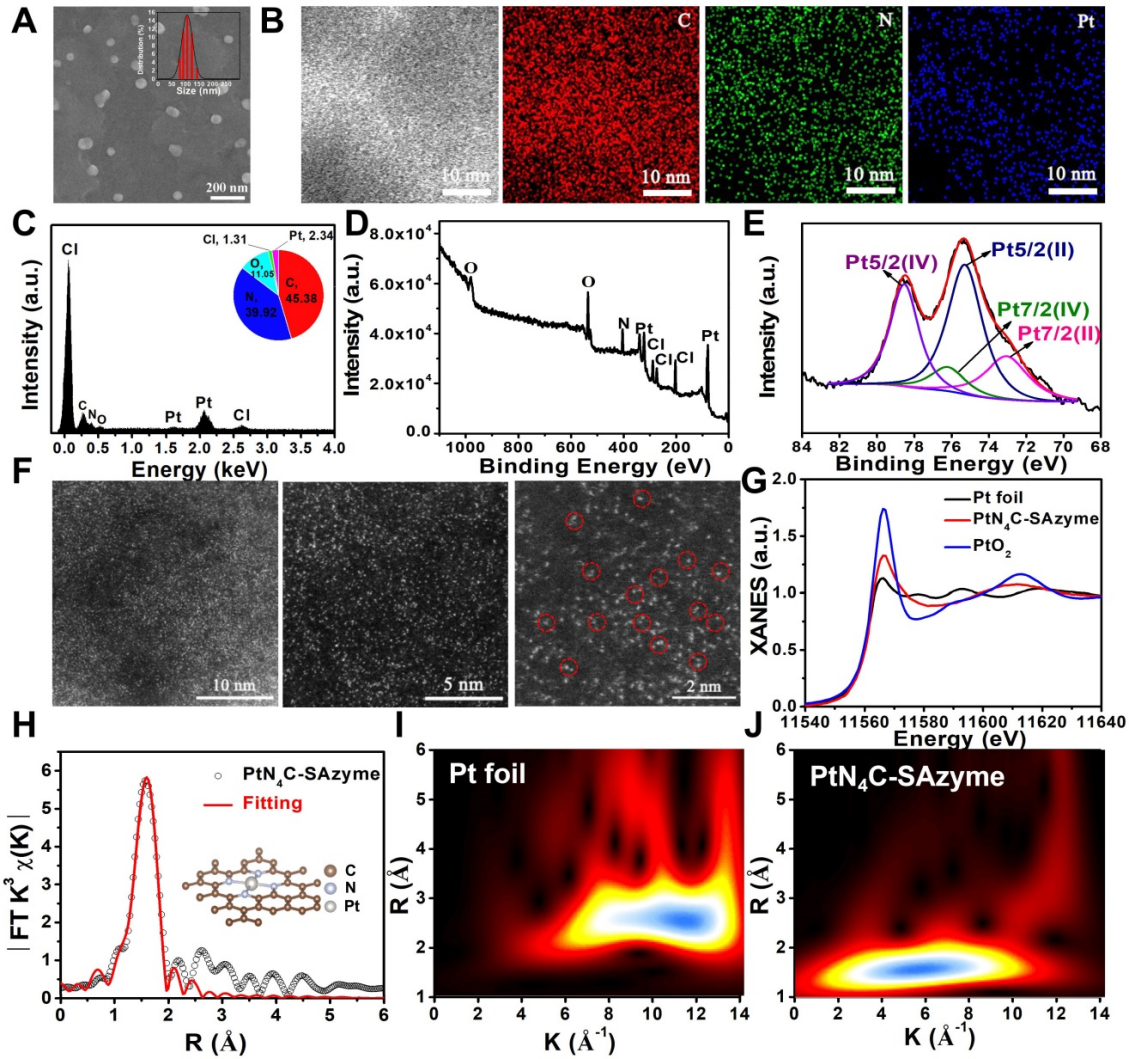
** Structural characterizations and atomic structural analysis of PtN_4_C-SAzyme.** SEM image (A) and the corresponding element mapping (B) of PtN_4_C-SAzyme; (C) EDS spectra recorded from the whole area of PtN_4_C-SAzyme. XPS spectrum of PtN_4_C-SAzyme (D) and high-resolution Pt4f of PtN_4_C-SAzyme (E). (F) Aberration-corrected HAADF-STEM image of PtN_4_C-SAzyme with different magnification. (G) XANES spectra of Pt foil and PtN_4_C-SAzyme. (H) Fourier-transformed magnitudes of the experimental Pt K-edge EXAFS signals of PtN_4_C-SAzyme. Wavelet transform (WT) for the k^3^-weighted EXAFS spectra of (I) Pt foil and (J) PtN_4_C-SAzyme.

To substantiate our protocol, the PtN_4_C-SAzyme was successfully synthesized according to a reported strategy with a slight modification (**Scheme 1**) 68, 69. Representative scanning electron microscopy (SEM) indicated that the PtN_4_C-SAzyme exhibited uniform size and spheroid morphology (**Figure 1A**). Corresponding dynamic light scattering (DLS) measurement confirmed that PtN_4_C-SAzyme possessed admirable dispersion with an average hydrodynamic diameter of about 110 nm (**Figure 1A**), which was facilitated to uptake and penetrate nanomedicines at tumor site due to the enhanced permeability and retention (EPR) effect 70. Furthermore, the element mappings and energy-dispersive spectroscopy (EDS) spectrum showed the Pt, N and C atoms were homogeneously distributed over the entire architecture of PtN_4_C-SAzyme (**Figure 1B**), and Pt and N content in PtN_4_C-SAzyme was quantified to be 2.34 and 39.92 wt %, respectively (**Figure 1C**). Consistently, X-ray diffraction (XRD) pattern of PtN_4_C-SAzyme disclosed no observable Pt nanoparticles and/or nanoclusters or characteristic crystal peaks, thereby excluding the formation of Pt-based crystalline phases and illustrating the successful formation of PtN_4_C-SAzyme (**Figure S1**). In addition, Fourier transform infrared spectroscopy (FT-IR) spectra (**Figure S2**), UV/vis absorption spectra (**Figure S3**) and ζ potentials (**Figure S4**) also demonstrated the successful preparation of PtN_4_C-SAzyme. Moreover, the existence of Pt, N, C elements and Pt-N interaction was further substantiated by X-ray photoelectron spectroscopy (XPS) analysis (**Figure 1D**). Given the importance of metal valence state in tumor catalytic therapy, the high-resolution Pt 4f XPS spectrum was further identified. As shown in **Figure 1E**, Pt-Pt bonds located at 71.2 eV and 74.5 eV was absence, and the deconvolution of XPS spectrum of PtN_4_C-SAzyme in Pt 4f region showed two characteristic peaks located at 78.5 eV and the peaks at 75.4 eV, respectively, owing to the coexistence of Pt^4+^ and Pt^2+^ 71, which was consistent with XRD result and indicated the successful generation of Pt-N_x_ sites in three-dimensional skeleton. It is reported that low metal valence state will facilitate the decomposition of H_2_O_2_ to ∙OH generation under acidic conditions, while the high valence state will promote the generation of O_2_ by the oxidation of H_2_O_2_ 7,17. Therefore, because of the coexistence of their mixed valance state, PtN_4_C-SAzyme is greatly anticipated to be a nanozyme with superior peroxidase-like and catalase-like activity for tumor-specific catalytic therapy.

To verify whether the isolated Pt atoms are atomically dispersed in PtN_4_C-SAzyme, aberration-corrected high-angle annular dark-field scanning transmission electron microscopy (HAADF-STEM) characterizations were employed and bright dots highlighted by red circles were recognized to be Pt single atoms in the PtN_4_C-SAzyme (**Figure 1F**), directly visually and intuitively demonstrating the existence of isolated Pt atoms. To further validate the local structure and coordination environment of the PtN_4_C-SAzyme at an atomic level, synchrotron radiation-based X-ray absorption near-edge structure (XANES) and extended X-ray absorption fine structure spectroscopy (EXAFS) measurements were carried out at Pt K-edge. The near-edge absorption energy position of PtN_4_C-SAzyme was located between that of reference samples Pt foil and PtO_2_ (**Figure 1G**), indicating that the atomically dispersed Pt species carried a positive charge between Pt^0^ and Pt^4+^, in accordance with XPS Pt4f spectrum mentioned above. Moreover, Fourier-transformed k^3^-weighted extended XAFS (FT-EXAFS) spectrum of PtN_4_C-SAzyme at Pt K-edge presented a main peak at 1.59 Å, which was drastically different from Pt-Pt bond (2.6 Å) (**Figure 1H**) 72-76, further strongly excluding the existence of Pt-based crystal and confirming the formation of atomically dispersed Pt-N_x_ sites structures in PtN_4_C-SAzyme. Furthermore, wavelet transform (WT) of the k^3^-weighted EXAFS spectra were also obtained in **Figure 1I&J**. As displayed in the contour plot of standard Pt foil, the maximum intensities at ~11.8 was contributed to Pt-Pt bonding. However, an intensity maximum of ~6.1 Å in the PtN_4_C-SAzyme is accordant to the Pt-N bonding. Meanwhile, the corresponding EXAFS fitting parameters were listed in **Table S1**. The obtained surrounding coordination atom (N) of Pt in the PtN_4_C-SAzyme was about 4 and the bond length between Pt and N atoms was 2.01. Therefore, all these results indicated the Pt in the PtN_4_C-SAzyme was atomically dispersion.

**Figure 2:**
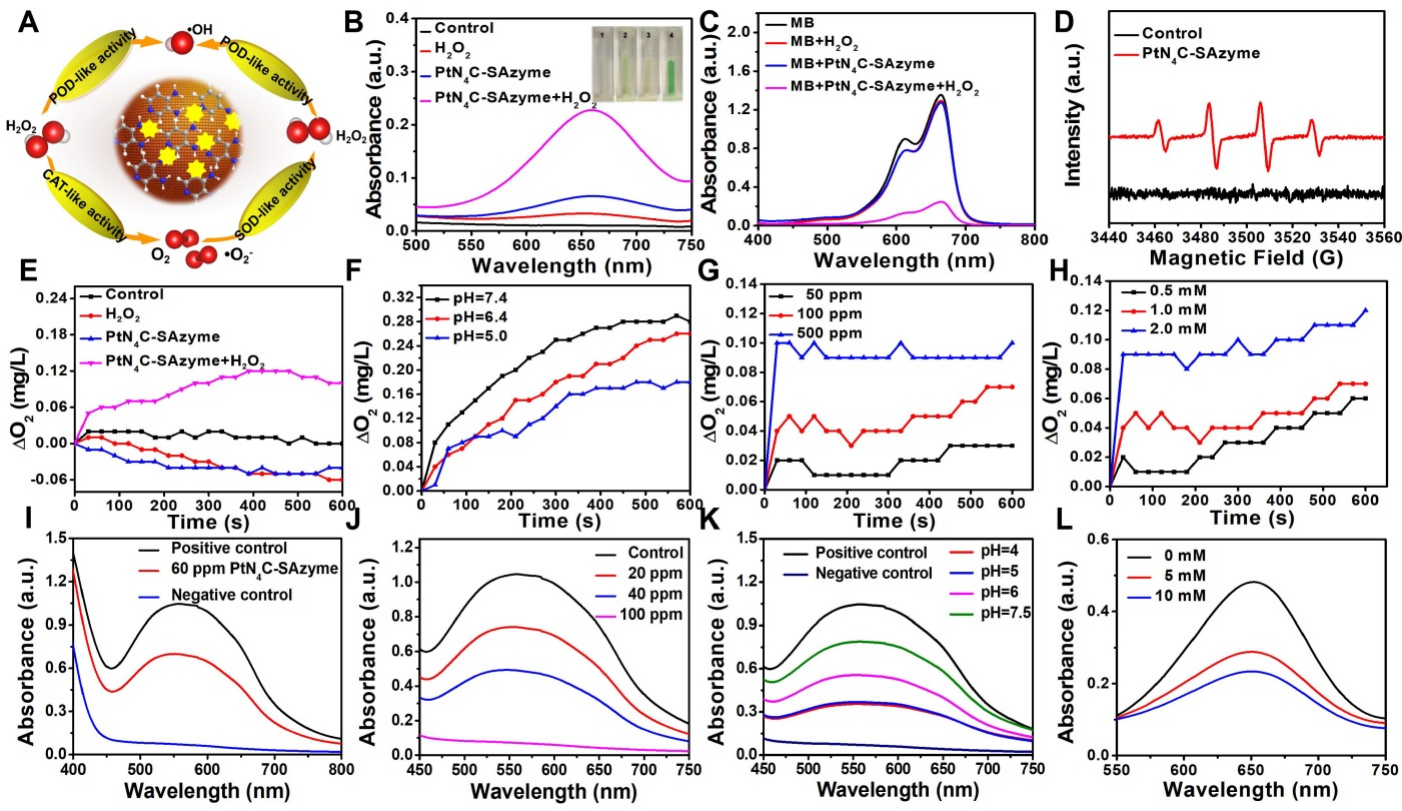
** Multiple enzyme-like activities of PtN_4_C-SAzyme for tumor microenvironment regulation.** (A) Schematic presentation of superoxide dismutase-like, peroxidase-like and catalase-like activities of PtN_4_C-SAzyme; (B) Peroxidase-like activities of PtN_4_C-SAzyme; (C) The degradation of MB in different solutions by the PtN_4_C-SAzyme mediated Fenton-like reaction. (D) EPR spectra for the detection of ·OH from H_2_O_2_ and PtN_4_C-SAzyme+H_2_O_2._ (E) O_2_ generation in different systems in the absence or presence of PtN_4_C-SAzyme. (F-H) The catalase-like activity of the PtN_4_C-SAzyme is dependent on pH, samples and H_2_O_2_ concentration. (I) Superoxide dismutase-like of PtN_4_C-SAzyme. (J) Catalytic conversion of superoxide radicals by different concentration of PtN_4_C-SAzyme. (K) UV-visible absorption of oxidized NBT in different conditions. (L) UV-vis spectra of the peroxidase-like activity of PtN_4_C-SAzyme with and without KSCN.

Subsequently, the mixed valence state of PtN_4_C-SAzyme inspired us to investigate its multiple enzyme-like catalytic functions for biocatalytic cascade reactions, which plays an important role in the metabolic processes and cancer therapy (**Figure 2A**). In the first place, the ·OH-generating peroxidase-like activity of PtN_4_C-SAzyme was evaluated by the catalytic oxidation of 3, 3′, 5, 5′-tetramethylbenzidine (TMB) to form the blue-colored oxidized TMB (oxTMB). As depicted in **Figure 2B**, in the presence of PtN_4_C-SAzyme and H_2_O_2_, TMB could be effectively oxidized and showed an obvious absorbance increase at the characteristic peak of 652 nm, whereas with PtN_4_C-SAzyme or H_2_O_2_ alone, the characteristic absorption of oxTMB disappeared, indicating no oxidation reaction was occurred and uncovering the superior peroxidase-like activity of PtN_4_C-SAzyme. Moreover, the ∙OH generation capacity by PtN_4_C-SAzyme was further monitored through the degradation of methylene blue (MB) (**Figure 2C**). After incubation with PtN_4_C-SAzyme with H_2_O_2_, the MB content significantly decreased by 83.0 %, while no obvious change was observed in PtN_4_C-SAzyme or H_2_O_2_ alone, indicating the effective ·OH generation was driven by the PtN_4_C-SAzyme-mediated Fenton-like reaction. Meanwhile, we also confirmed the existence of the active intermediate ·OH generation during the catalytic processes by electron spin resonance (ESR) analysis. As described in **Figure 2D,** the signal of DMPO/·OH significantly increased in the presence of PtN_4_C-SAzyme. And once adding the ·OH scavenger of isopropanol, the absorbance signal of SAzymes markedly declined, further substantiating that ·OH participated in the catalytic processes (**Figure S5**). Furthermore, to elucidate the peroxidase-like catalytic mechanism of PtN_4_C-SAzyme, the H_2_O_2_ decomposition efficiency was determined in the catalytic reaction. The result showed that the concentration of H_2_O_2_ significantly decreased once in the presence of PtN_4_C-SAzyme (**Figure S6**), manifesting that PtN_4_C-SAzyme with high peroxidase-like catalytic activities had the great potential to decompose H_2_O_2_ and generate ∙OH. Apart from these, some factor-dependent behaviors like concentration, H_2_O_2_, temperature, and pH were also investigated (**Figure S7**). And the catalytic kinetics of PtN_4_C-SAzyme exhibited a typical Michaelis-Menten kinetics for H_2_O_2_ and TMB substrates under the optimal pH and temperature, respectively (**Figure S8-9**). Subsequently, to investigate the PtN_4_C-SAzyme possessing favorable catalase-like activity, we measured H_2_O_2_-triggered O_2_-evolving in PtN_4_C-SAzyme by a dissolved oxygen meter, which is crucial for chemoradiotherapy of hypoxia tumor. As expected, the PtN_4_C-SAzyme exhibited an obvious catalase-like activity in the presence of H_2_O_2_ (**Figure 2E&S10**). Although the content of dissolved oxygen (DO) decreased with the decrease of pH value, there was still much higher than that of SAzymes or H_2_O_2_ alone at pH 5.0 (**Figure 2F&S11**), implying that the PtN_4_C-SAzyme could trigger the decomposition of H_2_O_2_ to produce O_2_ under tumor acidic microenvironment and facilitate to alleviate tumor hypoxia. In addition, the catalase-like activity of PtN_4_C-SAzyme also exhibited samples and H_2_O_2_ concentration-dependent manner (**Figure 2G&H**). According to the above results, we could conclude that PtN_4_C-SAzyme had great promise for tumor CDT and chemoradiotherapy catalytic therapy by their intrinsic H_2_O_2_-responsive peroxidase- and catalase-like activities.

As we all known, although the TME is characterized by a low pH value, hypoxia, overexpressed GSH and H_2_O_2_ compared with normal physiological condition, the level of inherently endogenous H_2_O_2_ (50-100×10^-6^ M) is insufficient to generate substantial ·OH and O_2_ for achieving tumor satisfactory CDT and chemoradiotherapy therapeutic effect. Therefore, we anticipate our PtN_4_C-SAzyme possesses superoxide dismutase activity for promoting superoxide anions (O_2_^·-^) transformation into H_2_O_2_ by disproportionation catalysis, thereby realizing continuous H_2_O_2_-supplement for potentiating their CDT and chemoradiotherapy. Unexpectedly, apart from the peroxidase- and catalase-like activities, PtN_4_C-SAzyme also exhibited the capacity of transforming O_2_^·-^ generated from chemoradiotherapy into H_2_O_2_ measured by the classic nitroblue tetrazolium (NBT) chromogenic method, achieving the cyclic accumulation of H_2_O_2_ at tumor site and subsequently decomposition into ∙OH and O_2_. As displayed in **Figure 2I**, O_2_^·-^ in the positive control was generated by irradiation riboflavin and Lmethionine with UV, which could decrease NBT to a water-soluble formazan dye with a strong UV-vis absorbance signal at the characteristic peak of 560 nm. Once PtN_4_C-SAzyme was added, the absorbance signal decreased significantly. Moreover, the disproportionation of PtN_4_C-SAzyme to O_2_^·-^ also exhibited concentration and pH-dependent (**Figure 2J-K and S12**). With the increase of the SAzyme concentration, the disproportionation of PtN_4_C-SAzyme to O_2_^•-^ was further increased (**Figure 2J**). Notably, PtN_4_C-SAzyme exhibited better enzyme-like activity at lower pH conditions (**Figure 2K**), which facilitates the generation of hydrogen peroxide in the acidic tumor microenvironment to enhance tumor therapy. Importantly, as a proof-of-concept, we also adopted KSCN to block Pt sites to confirm the active sites (**Figure 2L**). When in the presence of KSCN, the activity of SAzymes significantly decreased due to the chelation between single Pt atom and SCN-, indirectly revealing Pt-Nx sites act as their active sites. Therefore, all these results indicated that the PtN_4_C-SAzyme could act as three-enzymatic co-expression systems for achieving continuous H_2_O_2_ self-supplement and cyclic accumulation of substantial ∙OH and O_2_ under TME, so as to greatly boost therapeutic outcome for CDT and chemoradiotherapy of hypoxia tumor.

**Figure 3:**
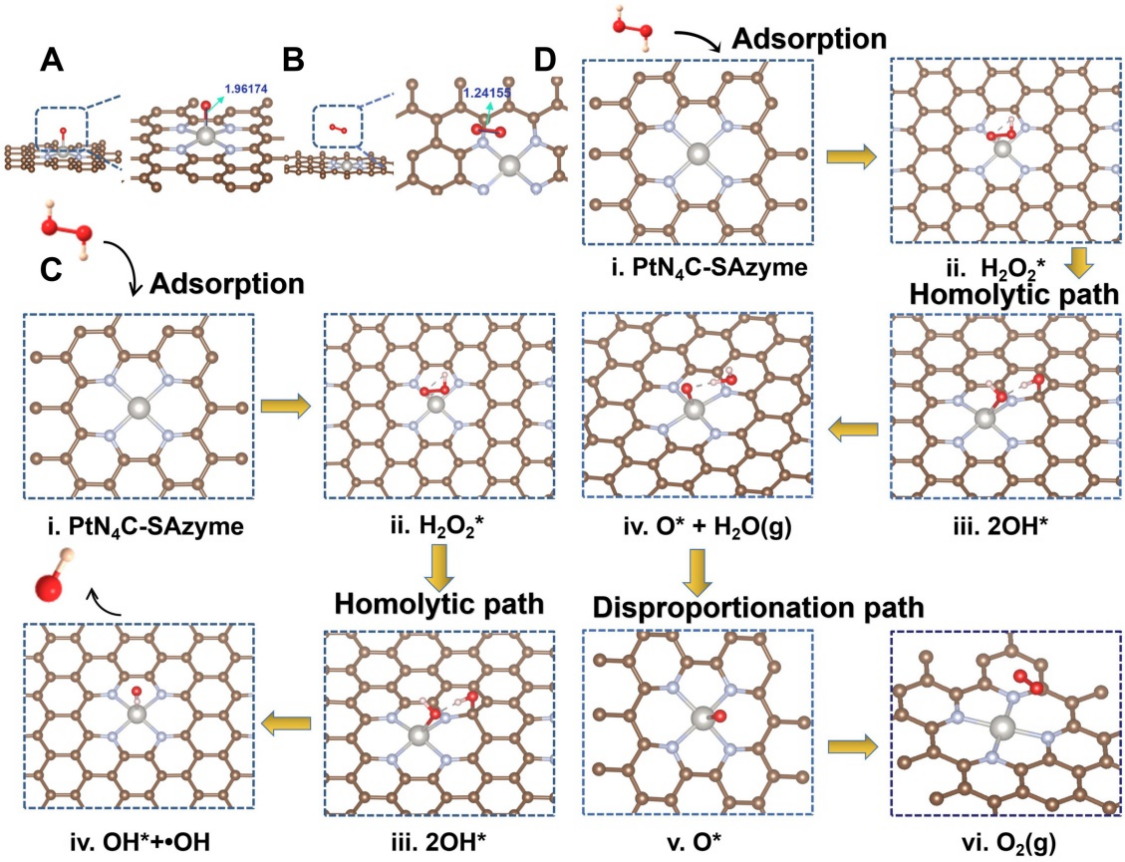
** Density functional theory (DFT) calculations of PtN_4_C-SAzyme.** Optimized structures of O (A) and O_2_ (B) species adsorbed on the surface of PtN_4_C-SAzyme, respectively. Interatomic distances are indicated in Å. Proposed catalytic mechanism schematics of peroxidase-like (C) and catalase-like (D) activities of PtN_4_C-SAzyme. The dark grey, light grey, gold, red and pink balls represent the Pt, N, C, O and H atoms, respectively.

To shed light on the origin of the multiple enzyme-like activities of PtN_4_C-SAzyme, plausible reaction mechanisms and reaction pathways as well as the free energy profiles of the confined Pt site are put forward by density functional theory (DFT) calculation. EXAFS fitting was firstly performed to support the quantitative structural configuration of Pt in the PtN_4_C-SAzyme sample with a coordination number of 4.0 (**Table S1**). Therefore, an optimized structure model of Pt-N_x_ active site embedded in the PtN_4_C-SAzyme matrix was established according to experimental characterization, accompany with a Pt-O bond length of 1.961 and 1.241 Å respectively after adsorption O and O_2_ on the PtN_4_C-SAzyme (**Figure 3A&B**). As revealed in **Figure 3C**, the geometrically optimized H_2_O_2_ molecule is initially adsorbed on the top of the Pt active centers (Pt-N_x_) of PtN_4_C-SAzyme (i), resulting in the adsorbed intermediates (ii) formation with a moderate adsorption energy (H_2_O_2_*) of 0.29 eV. The activated H_2_O_2_ molecule is subsequently cleaved to 2OH* (iii) by the single Pt sites (Pt^δ+^) via a homogeneous pathway (1.005 eV) rather than heterogeneous manner (1.423 eV) (**Figure S13**). Subsequently, the intermediates of a reactive hydroxyl radical (∙OH) and a hydroxyl group (OH*) were generated by a hydroxyl group desorption from the single Pt site (iv), accompanying with energy barrier of 0.42 eV, which is easily surmountable at room temperature. Finally, desorption of a ∙OH enables the overall regeneration of the SAzymes surface for activating another H_2_O_2_ molecule to generate ∙OH in the next cycle, resulting in a substantial reduction of the total Gibbs free energy and achieving their superior peroxidase-like activity (-0.65 eV) (**Figure S14**). In addition, to further gain insights into the catalase-like activity of PtN_4_C-SAzyme toward H_2_O_2_ oxidation, two possible reaction pathways were put forward by DFT calculation (**Figure S15**). The results indicated that reaction energy barrier for the homolytic pathway of H_2_O_2_* to 2OH* (1.005 eV) was lower than that for the heterolytic pathway of H_2_O_2_* to O* + H_2_O* (1.423 eV), suggesting the homolytic pathway was more kinetically favorable for H_2_O_2_* dissociation and O_2_ generation. Therefore, we adopted the homolytic pathway as a dominant pathway to investigate the reaction mechanism for the enhanced catalase-like activity of PtN_4_C-SAzyme. **Figure 3D** showed that the H_2_O_2_ molecule could be easily adsorbed on the confined Pt site (i) for forming an Pt-H_2_O_2_* intermediate (ii) and releasing two OH* molecule (iii), followed by the dissociation for generating O* and H_2_O molecule (iv) by disproportionation pathway with an energy barrier of -0.38 eV and releasing one H_2_O molecule (v) subsequently. Finally, the O* species of the Pt-O was active for the adsorption of another O* via the formation of an O_2_ molecule with an energy barrier of -3.87 eV. Therefore, the DFT calculations unambiguously identified that the Pt-N_x_ centers could play an important role in the adsorption and activation of H_2_O_2_, which were in agreement with the origin of generating ∙OH and O_2_ by their superior peroxidase- and catalase-like activity.

**Figure 4:**
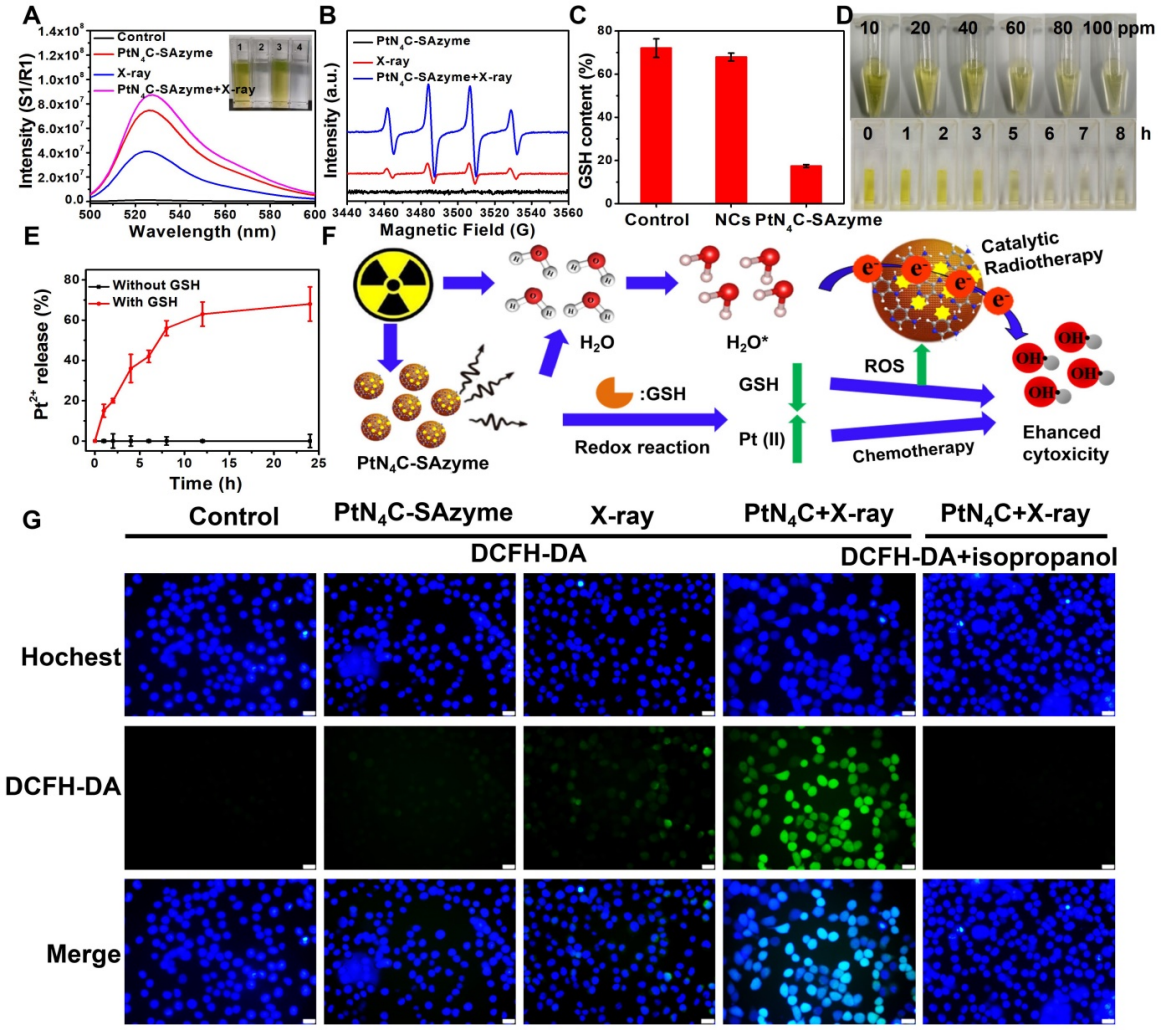
** PtN_4_C-SAzyme-enhanced tumor chemoradiotherapy by generation Pt^2+^ and ROS with the depletion of GSH**. (A) Fluorescence spectra of DCFH-DA mixed with PtN_4_C-SAzyme (100 μg/mL) exposed to X-ray. (B) EPR spectra for the detection of ·OH from PtN_4_C-SAzyme in the presence or absence of X-ray. (C) Relative content of GSH before and after addition of PtN_4_C-SAzyme and NCs. (D) Photographs of glutathione oxidation by recording the color change of the solution mixture with the changes in concentration and time. (E) Pt^2+^ release profiles of PtN_4_C-SAzyme with or without GSH. (F) Mechanism diagram of the reaction. (G) ROS generation of 4T1 cells with different treatments. The scale bar is 100 µm. Error bars were calculated by SD of three parallel samples.

Interestingly, apart from their multi-enzyme cascade biocatalytic activity for TME regulation and tumor CDT, we uncovered that the PtN_4_C-SAzyme also possessed favorable X-ray-triggered radiosensitization behavior and endogenous GSH-activated Pt^2+^ release capacity to boost their chemoradiotherapy effciency. In the first place, both 7'-Dichlorofluorescent yellow diacetate (DCFH-DA) and 5, 5-dimethyl-1-pyrroline N-oxide (DMPO) were employed as probes to monitor the generation of ROS in different systems. As depicted in **Figure 4A&B,** negligible fluorescence or characteristic signals were detected in the group of phosphate buffer, SAzyme solution or X-ray alone. However, the fluorescence intensity and characteristic signals of the PtN_4_C-SAzyme solution were significantly increased once in the presence of X-ray. This might be attributed to abundant Pt active sites and strong surface charge density in the surface of PtN_4_C-SAzyme for sufficient ROS generation by enhancing X-ray deposition to produce H_2_O^+^ and H_2_O* as well as ∙OH in the successive physico-chemical and chemical stages. Moreover, the deprivation of GSH by our SAzymes via the redox reaction was studied, which was facilitated to generate large amounts of ROS for killing the tumor. As displayed in **Figure 4C**, obviously decrease of GSH was induced after incubation with PtN_4_C-SAzyme, in comparison with that of N-doped carbon support (NCs). And the consumption of GSH by PtN_4_C-SAzyme also exhibited concentration- and time-dependent behaviors (**Figure 4D&S16**), which demonstrated that PtN_4_C-SAzyme could effectively promote GSH depletion through redox reaction. Meanwhile, we were surprised to find that the PtN_4_C-SAzyme could trigger Pt^2+^ release while eliminating GSH (**Figure 4E**), which not only could be used for chemotherapy effect and facilitate to overcome tumor drug-resistance, but also could be again converted to Pt^4+^ by the valence state variation of Pt, endowing the continuous deprivation of GSH. Therefore, a possible enhanced cytotoxicity mechanism of PtN_4_C-SAzyme may be described as follows (**Figure 4F**). The overexpression of GSH in the tumor can directly diminish the amount of ROS generation and thereby attenuate the cytotoxicity of cancer cells. Hearteningly, we found that the PtN_4_C-SAzyme can drastically enhance ROS levels through producing Compton and Auger electrons induced by PtN_4_C-SAzyme with the surrounding water or oxygen molecules and consuming GSH as well as promoting cytotoxic Pt^2+^ release by the redox reaction, thus effectively enhancing collaborative anti-tumor effect. Furthermore, the ROS-evolving properties of PtN_4_C-SAzyme under X-ray irradiation in cancer cells were also evaluated by utilizing the 2′, 7-dichlorodihydrofluorescein diacetate (DCFH-DA) probe. Indeed, the introduction of X-ray to PtN_4_C-SAzyme system resulted in significantly enhanced ROS generation in comparison with the control group (**Figure 4G**), effectively validating their remarkable radiosensitization capacity. Taken together, all these results demonstrated that PtN_4_C-SAzyme have potential to enhance synergetic CDT and chemoradiotherapy therapeutic efficiency, resulting from the TME-initiated multiple enzyme-like activities, X-ray-triggered ROS generation, GSH-depletion and Pt^2+^-releasing capabilities of PtN_4_C-SAzyme.

**Figure 5:**
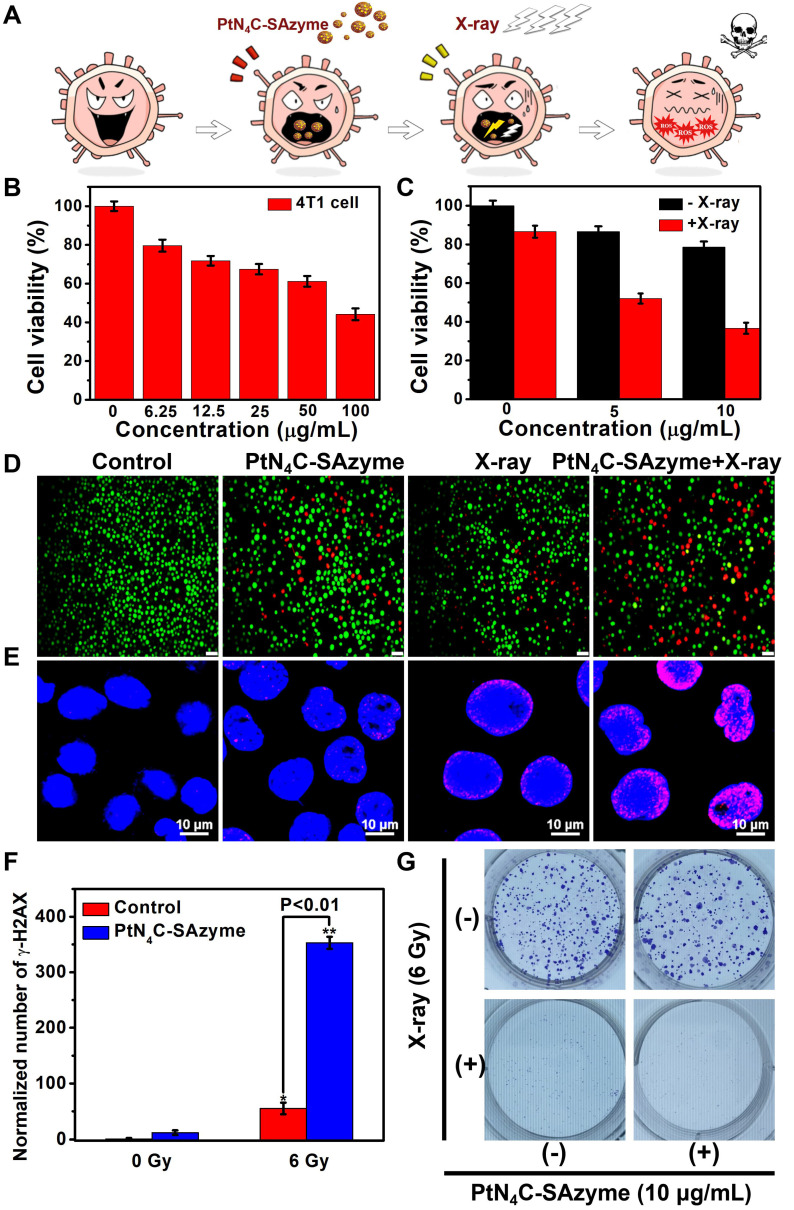
** PtN_4_C-SAzyme-enhanced CDT and chemoradiotherapy for increased apoptosis and DNA double-strand damage in 4T1 cells.** (A) Schematic illustration of 4T1 cell therapeutic outcome by combination PtN_4_C-SAzyme with X-ray. (B) Cell viability of 4T1 cell after treated with different concentrations of PtN_4_C-SAzyme. (C) Cell viability of 4T1 cells co-incubated with PtN_4_C-SAzyme in the presence or absence of X-ray. (D) Live/dead co-staining images of 4T1 cells in different solutions. Scale bar=20 μm. Representative fluorescence images (E) and corresponding normalized number (F) of γ-H2AX of DNA fragmentation and nuclear condensation induced by PtN_4_C-SAzyme (10 μg/mL, 2 mL) and/or X-ray radiation (6 Gy). (G) Colon formation of 4T1 cells after different treatments. Opposed to (-), (+) denotes X-ray was in the presence of in the group. Error bars were calculated by SD of three parallel samples. P values were based on the Student's test: *P < 0.05, **P < 0.01.

After proving the promising ∙OH generation capacity of PtN_4_C-SAzyme under X-ray irradiation in aqueous solutions, we further substantiated at the cellular level whether the X-ray-triggered ROS generation, GSH-depletion and Pt^2+^-releasing properties could effectively inhibit cancer cell growth and improve cell killing (**Figure 5A**). As illustrated in **Figure 5B**, their potential chemotherapy performance against 4T1 cells was first evaluated according to the standard cell counting Kit-8 (CCK-8) assay. Compared to untreated cells, the cell viabilities exhibited SAzymes concentration-dependent cytotoxicity. When the PtN_4_C-SAzyme concentration was 100 μg mL^-1^, the cell viabilities decreased to 42 %, which might be ascribed to the excellent Fenton-like Pt^2+^ delivery and GSH depletion of PtN_4_C-SAzyme under intracellular reduction environments. Furthermore, the introduction of X-ray into PtN_4_C-SAzyme system further promoted cancer cell killing and also exhibited an X-ray dose-dependent toxicity to 4T1 cells for chemoradiotherapy (**Figure 5C**). And the cell live/dead double staining assay indicated that the cell viability of PtN4C-SAzyme+X-ray group is remarkably lower than that of the PtN_4_C-SAzyme or X-ray alone, (**Figure 5D&S17**), confirming their distinguished synergistic capability of GSH-triggered Pt^2+^ release behavior and local radiation deposition for remarkable ·OH production of synergistic CDT and chemoradiotherapy. It is well known that the intracellular ROS burst may cause multiple damages in chemoradiotherapy, particularly inducing DNA double-strand breaks (DSBs) for causing cell apoptosis or programmed cell death. Therefore, we further explored the underlying mechanisms of PtN_4_C-SAzyme-mediated enhanced radiosensitization by detecting DNA DSBs with labeling γ-H2AX, a marker of double-stranded DNA breakage with high specificity and accuracy. The results displayed that significantly increased detectable γ-H2AX immunofluorescent spots were observed in the presence of X-rays (6 Gy), particularly in the PtN_4_C-SAzyme group (**Figure 5E&S18**). However, only few of intranuclear spotty fluorescence from γ-H2AX were discovered without irradiation. And the γ-H2AX immunofluorescent spots of the PtN_4_C-SAzyme increased by approximately 91.67 % and 84.42 % compared with PBS and PtN_4_C-SAzyme, respectively, which were calculated via counting the red fluorescent foci (**Figure 5F**), demonstrating PtN_4_C-SAzyme possessed the admirable therapeutic activity and can cause severe oxidative stress in cancer cells. It was worth noting that short-term CCK-8 assay was uncommon to investigate survival of irradiated cells, clonogenic assay was further conducted to evaluate the enhanced radiotherapy efficiency of the PtN_4_C-SAzyme for accurate comparison. As shown in **Figure 5G**, the colonies were densely packed in the control group. However, the surviving fractions of the PtN_4_C-SAzyme groups were relatively lower, indicating that the PtN_4_C-SAzyme exhibited chemotherapy effect at the experimental concentration and had some effect on cell proliferation. Specifically, the cell survival fraction significantly decreased to 2.7 % after the X-ray irradiation treatment (6 Gy) combined with PtN_4_C-SAzyme, indicating that the PtN_4_C-SAzyme was excellent for radiosensitization. Consequently, all these data confirmed that TME-activatable PtN_4_C-SAzyme with remarkable H_2_O_2_ self-supplement, O_2_-evolving, and GSH depletion-induced Pt^2+^ delivery capacities had great potential application for tumor CDT and chemoradiotherapy.

**Figure 6:**
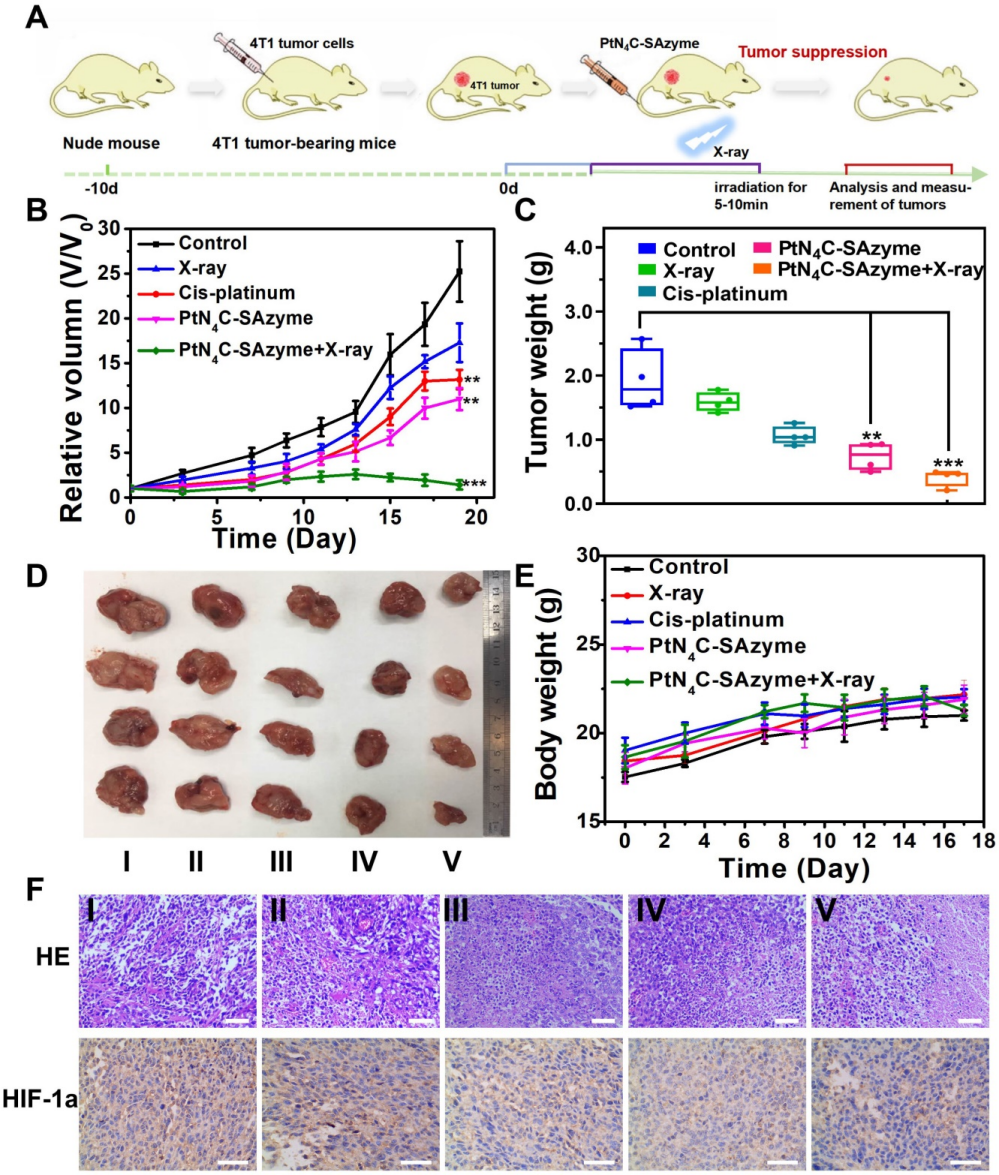
***In vivo* X-ray-enhanced PtN_4_C-SAzyme-based tumor synergistic catalytic therapy against 4T1 tumor xenografts.** (A) Schematic illustration of 4T1 tumor xenograft establishment and therapeutic outcome. Tumor volume (B) and tumor weight changes (C) of 4T1-tumor-bearing mice after different treatments at 19 days. Tumor photographs (D) and weight (E) of mice in different groups after 19 days of treatments. (F) H&E and HIF-1α staining assay of tumor tissues in different groups. Scale bar=50 μm. P values were based on the Student's test: *P < 0.05, **P < 0.01.

Encouraged by the intriguing catalytic-therapeutic efficacy of PtN_4_C-SAzyme *in vitro*, we inferred PtN_4_C-SAzyme might also have a considerable synergistic CDT and chemoradiotherapy efficacy *in vivo*. In order to prove this point, their enhanced CDT and chemoradiotherapy *in vivo* was investigated in the 4T1 cancer xenograft model using the BALB/c nude mice (**Figure 6A**). The 4T1 tumor-bearing nude mice were randomly divided into five groups, including (i) PBS, (ii) X-ray irradiation, (iii) cisplatin, (iv) PtN_4_C-SAzyme, (v) PtN_4_C-SAzyme and X-ray irradiation. As indicated in **Figures 6B-D**, tumor growth in mice treated with PtN_4_C-SAzyme was significantly suppressed compared with other control groups, especially irradiation with X-ray irradiation, which could be attributed to the X-ray-responsive PtN_4_C-SAzyme-induced severe oxidative stress. In addition, due to the drug-resistance of cisplatin, the volume of tumor in the cisplatin group was slightly larger than the PtN_4_C-SAzyme group, manifesting that our PtN_4_C-SAzyme have remarkable chemotherapy effect for overcoming drug-resistance and suppressing tumor growth. The striking anti-tumor efficacy of PtN_4_C-SAzyme may be beneficial from the continual accumulation of H_2_O_2_, overexpressed GSH depletion-triggered Pt^2+^ release and O_2_ evolving-induced tumor hypoxia alleviation for *in situ* generating numerous ROS. Following that, the toxicology of PtN_4_C-SAzyme was systematically investigated to guarantee their safe bioapplication. Encouragingly, no apparent systemic toxicity induced by PtN_4_C-SAzyme in the presence or absence of X-ray irradiation was observed, as depicted in the major organs, blood biochemistry and hematology analysis (**Figure S19-21**). In addition, all these mice in each group exhibited negligible weight fluctuations (**Figure 6E**), proving the high biocompatibility of PtN_4_C-SAzyme. The pathological examination of tumors via hematoxylin and eosin (H&E) staining and Hypoxia-inducible factor 1α (HIF1-α)-mediated immunohistochemical analysis further demonstrated PtN_4_C-SAzyme combination with X-ray could sharply augment cancer cell apoptosis by inducing severe oxidative stress and alleviate tumor hypoxia (**Figure 6F**). Therefore, these combined results demonstrated that the utilization of PtN_4_C-SAzyme could greatly treated 4T1 tumors with enhanced efficacy and minimized systemic toxicity.

## Conclusion

In summary, we have successfully developed a specific TME-activated SAzymes-based with parallel cascaded catalytic performance to break through the challenge for intensive CDT and chemoradiothearpy. At the specific microenvironment of tumor, the SAzyme could boost substantial ∙OH generation and O_2_ self-sufficiency for magnifying ROS-involving CDT and O_2_-dependent chemoradiothearpy effect by their favorable multiple enzyme-like catalytic activities as well as X-ray deposition capacity. Simultaneously, sustainable GSH consumption and elevated Pt^2+^ release could be achieved *in situ* of tumor by the self-cyclic valence alteration of Pt (IV) and Pt (II) for overwhelming the anti-oxidation defense effect and alleviating drug-resistance of hypoxia tumor. More significantly, the superoxide dismutase-like activity of SAzyme endowed H_2_O_2_ cyclic accumulation, which could in turn react with PtN_4_C-SAzyme to achieve the cyclic blowout of ∙OH and O_2_, so as to generate a “key” to unlock the multiple enzyme-like activities of SAzymes for tumor-specific cascaded catalytic CDT and chemoradiothearpy. Overall, we anticipate that this TME-inspired strategy will be a strong tool to treatment of various diseases and provide new perspective for us to broaden the biomedical applications of SAzymes.

## Experimental Section

### Materials

Cisplatin, 3, 4-diaminopyridine (DAP), 3, 3′, 5, 5′-tetramethylbenzidine (TMB), isopropanol, and potassium thiocyanate (KSCN) were purchased from Aladdin Reagent CO, Ltd. (Shanghai, China). Calcein AM and propidium iodide (PI) were obtained from Sigma-Aldrich (USA). Hydrogen peroxide (H_2_O_2_, 30 %) was ordered form Jinshan chemical reagent Co. Ltd (Chengdu, China). Reduced glutathione (GSH) was obtained from Nanjing jiancheng institute of biological engineering. 4 % paraformaldehyde was bought from Labgic Technology Co, Ltd (Beijing, China). Xanthine (X), Xanthine oxidase (XO), Methylene blue (MB) and Nitro-blue tetrazolium (NBT) were purchased from Sigma-Aldrich, USA. Reactive oxygen species assay kit based on 2, 7-Dichlorodi-hydrofluorescein diacetate (DCFH-DA) was acquired from Beyotime Biotechnology Co, Ltd. Live/Dead dye for viability/cytotoxicity assay was acquired from enzyme-linked Biotechnology Co., Ltd (Shanghai, China). All chemicals were utilized as purchased without further purified.

### Characterization

The morphology and composition of samples were characterized by using transmission electron microscope (TEM, FEI, Tecnai, G2 F20, USA) by dropping PtN_4_C-SAzyme samples onto a carbon-coated copper grids and drying them naturally before imaging. SEM images were acquired on a Zeiss Sigma 500 FE-SEM (Carl Zeiss AG, Oberkochen, Germany) at a working voltage of 10 kV and working current of 10 μA after 60 s of platinum coating. The X-ray absorption spectra (XAS) including X-ray absorption near-edge structure (XANES) and extended X-ray absorption fine stucture (EXAFS) of the samples at Pt K-edge (11564 eV) were collected at the Singapore Synchrotron Light Source (SSLS) center, where a pair of chanel-cut Si (111) crystals was used in the monochromator. The Pt K-edge XANES data were recorded in a transmission mode. Pt foil was used as reference. Fourier-transform infrared spectra (FTIR) of PtN_4_C-SAzyme was measured on an FT-IR spectrometer (IR 200, Thermo Fisher Scientific, US) with KBr particles in the range region of 4000-500 cm^-1^. The Raman spectrum of the PtN_4_C-SAzyme samples was recorded on a Raman spectrophotometer (Invia Reflex, Renishaw, UK). The optical characterizations of PtN_4_C-SAzyme were performed using a UV-vis spectrometer (UV-6100, Mapada, China) with a 1 cm cuvette. X-ray photoelectron spectroscopy (XPS) analysis was monitored using an ESCALAB 250 X-ray photoelectron spectrometer. X-ray diffractometry using Cu-Kα radiation (λ=0.15405 nm) was used to obtain crystallographic information. Zetasizer (Malvern, UK) was used to acquire the size distribution and ζ-potential of the as-synthesized PtN_4_C-SAzyme. Fluorescence spectra were obtained on a dual-FL luminescence spectrometer (HORIBA, Japan). The cells images were recorded using an inverted fluorescence microscope (Axio Vert.A1, Zeiss, Germany). The concentration of Pt^2+^ was quantitated using the inductively coupled plasma mass spectrometry (ICP-MS, Thermo Elemental X7).

### Synthesis of prodrug

The described method was according to a reported strategy by Jiang et al 68, 69. In detail, dried cis-[PtCl_2_(NH_3_)_2_] (0.4 g, 0.6 mmol) was suspended in water (14 mL) and a 10-fold excess of 30 % H_2_O_2_ ( 14 mL, 6 mmol) was then added, and the mixed system was reacted at 50 °C for 1 h. The obtained yellow solution was recrystallized, extracted and filtered to obtain crystals. Then, the crystals were washed sequentially with cold water, ethanol and ether, and finally dried in a desiccator to obtain a yellow solid powder (cis-[PtCl_2_(OH)_2_(NH_3_)_2_]). Whereafter, 0.2 g of the yellow solid powder obtained above (cis-[PtCl_2_(OH)_2_(NH_3_)_2_]) was dissolved in HCl (5 mL, 10 mol/L) at 50 °C and stirring for 1 h. After that, the above reaction system was moved away for continually stirring for 8 h at room temperature again to obtain a light yellow cisplatin precursor drug solution (cis-PtCl_4_(NH_3_)_2_), which was sealed and stored at room temperature.

### Synthesis of PtN_4_C-SAzyme

The method was according to a reported strategy with a slight modification 68, 69. Briefly, 25 mL of water and 0.13 g of 3, 4-diaminopyridine (DAP) were added into the previously obtained yellowish solution (cis-PtCl_4_(NH_3_)_2_) and the reaction was stirred for 24 h at 37 °C. After cooling down to room temperature, the resulting light red solution was acquired after purification through dialysis bag with cut-off 12 kDa MW for 5 h to remove the impurities and finally freeze-dried to obtain a dried sample powder for further use.

### Atomic structure analysis of PtN_4_C-SAzyme

The obtained extended X-ray absorption fine structure (EXAFS) data were manipulated according to a standard procedure using the ATHENA model implemented in the IFEFFIT software packages 71. Subsequently, the k3-weighted EXAFS spectra was acquired by the background at the post-edge from the overall absorption and then normalized with regard to the edge-jump step. The k3-weighted χ(k) data at the Pt L3-edge were then Fourier transformed into real (R) space by using a hanning windows (dk = 1.0 Å^-1^) to separate the EXAFS contributions from the various coordination shells. To acquire the quantitative structural parameters around the central atom, least-squares curve parameter fitting was conducted by using the ARTEMIS module of IFEFFIT software packages.

### Density functional theory (DFT) calculation

All the calculations were conducted within the framework of the density functional theory (DFT) as implemented in the Vienna Ab initio Software Package (VASP 5.3.5) code using the Perdew-Burke-Ernzerhof (PBE) generalized gradient approximation of the projected augmented wave (PAW) approach 77. The cutoff energy of the plane-wave fundamental was set at 400 eV. The Brillouin zone of the surface unit cell was simulated by a Monkhorst-Pack (MP) grid, and the k-point grid was used for structural optimization of Pt-N-doped graphene catalysts. The catalyst surface was decided by a 3×3×1 Monkhorst-Pack grid. The electronic self-consistent iteration and force convergence criteria were set to 10^-5^ eV and 0.01 eV/Å, respectively. In this work, we constructed a 3×2 catalyst surface supercell including 1 atomic layer to simulate Pt-N-doped graphene catalysts. A vacuum layer of 12 Å was introduced to prevent interactions between periodic images. The absorption energy (E_ads_) of the surface species is defined by E_ads_=E_total_-E_surface_-E_species_, where E_total_ denotes the total energy absorbed by the catalyst surface, Esurface denotes the energy of the empty surface, and Especies denotes the energy of the surface species in the gas phases.

### Peroxidase-like activity of PtN_4_C-SAzyme

The peroxidase-like activity of PtN_4_C-SAzyme was determined by UV-vis spectrometer (UV-6100, Mapada, China) after 30 min reaction. The absorbance of oxTMB at the characteristic peak of 652 nm was recorded by a UV-vis spectrometer for a certain reaction time to investigate the peroxidase-like activity of PtN_4_C-SAzyme. Four groups were divided: (i) Control, (ii) H_2_O_2_, (iii) PtN_4_C-SAzyme, (iv) PtN_4_C-SAzyme+H_2_O_2_. Typically, a quantified PtN_4_C-SAzyme, TMB (0.5 mM), and H_2_O_2_ (50 mM) were added to the NaAc buffer (pH = 4), successfully. To explore the optimal conditions of TMB oxidation by PtN_4_C-SAzyme, a range of temperatures (25, 30, 35, 40, 50, 60, 70 °C) and pH values as well as various H_2_O_2_ concentrations were used for the reactions under all other same conditions.

The apparent kinetic parameters were calculated by the Lineweaver-Burk double reciprocal equation derived from the Michaelis-Menten equation:



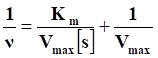



where v is the initial velocity, V_max_ is the maximal reaction velocity, [s] is the concentration of substrate and K_m_ is the Michaelis constant. The steady-state kinetic assays were performed at NaAc reaction buffer solution with PtN_4_C-SAzyme solution (100 μg/mL), H_2_O_2,_ and TMB. To explore this mechanism, the Michaelis-Menten constants were obtained from adjusting different concentrations of TMB and H_2_O_2_ under the standard reaction conditions described above. In addition, MB was also used as a probe to investigate the ∙OH generation by the Fenton-like reaction of PtN_4_C-SAzyme. In the first place, MB (10 μg/mL), PtN_4_C-SAzyme (100 μg/mL) and H_2_O_2_ (10 mM) were added sequentially in NaHCO_3_/5 % CO_2_ buffer solution (25 mM), and then the mixture stood still at 37 °C for 30 min protected from light. Then, the ·OH-induced decomposition of MB was detected by variation in absorption at 665 nm.

### Catalase-like activity of PtN_4_C-SAzyme

The catalase-like activities of PtN_4_C-SAzyme were measured by recording the generated oxygen at room temperature using a portable dissolved oxygen meter. In a typical test, 100 μg/mL of PtN_4_C-SAzyme (5 mL) and 100 μL 30 % H_2_O_2_ (0.5, 1, 2 mM) solution was added into the 44.9 mL of buffer solution (0.1 M NaOAc buffer, pH = 7.0), successively. The content of dissolved oxygen was monitored by using a dissolved-oxygen meter over 10 min (mg/L).

### Superoxide dismutase-like activity of PtN_4_C-SAzyme

The superoxide dismutase-like activities of PtN_4_C-SAzyme were evaluated by the classic nitroblue tetrazolium (NBT) chromogenic method. An O_2_^·-^-sensitive NBT probe was used to assess the superoxide dismutase-like activity of PtN_4_C-SAzyme. First, different concentrations of PtN_4_C-SAzyme were incubated with X (1 mM) and XO (0.05 U/mL) in Tris-HCl buffer (0.1 M, pH = 7.5) at 37 °C for 5 min. Then, NBT (100 μg/mL) was incorporated into the mixed solution and their absorbance at the characteristic peak of 550 nm was continuously monitored over 5 min using a UV-vis spectrometer. The complete reaction assembly was closed in a foil-lined box. Saline was used as a negative control and O_2_^·-^-sensitive NBT solution without PtN_4_C-SAzyme was used as a positive control. The inhibition rate was computed by the following formula: inhibition (%) = [(A_0_-A)/A_0_] × 100 %, where A_0_ is the absorption of the control and A is the absorption of the sample.

### GSH and H_2_O_2_ consumption

Extracellular GSH consumption was measured by a Glutathione Reductase Assay Kit with DTNB (Nanjing Jiancheng Bioengineering Institute, China). Firstly, PBS solution (pH = 7.4) containing PtN_4_C-SAzyme (100 μg/mL) and GSH (50 μM) were coincubated in a shaker at 25 °C for different times (1, 2, 3, 4, 5, 6, 7, 8 h). Afterward, these mixtures were centrifuged at 12000 rpm for 15 min to obtain the corresponding supernatants. The absorbance of corresponding supernatants solution was measured by a UV-vis spectrometer at 412 nm. In addition, the H_2_O_2_ concentration was evaluated using the H_2_O_2_ Assay Kit (Nanjing Jiancheng Bioengineering Institute, China). In brief, two groups were divided, which included H_2_O_2_ and PtN_4_C-SAzyme + H_2_O_2_. 0.1 mL of SAzyme sample or standard was combined with 1 mL of reagent I and reagent II, respectively and the absorption of mixed solution was measured at 405 nm.

### Detection of reactive oxygen species produced by PtN_4_C-SAzyme under X-ray irradiation

Firstly, 0.5 mL of DCFH-DA in DMSO was combined with 2 mL of NaOH (0.01 M) at room temperature and the mixtures were chemically hydrolyzed to DCFH. After 30 min, 10 mL of phosphate buffer (PBS, 25 mM, pH 7.2) was inserted and the resulted DCFH solution was covered with aluminum foil and placed on ice for further experiments. Subsequently, PtN_4_C-SAzyme (100 µg/mL) was mixed with DCFH (10 µM) solution in the absence or presence of GSH (1 mM) and then irradiated with X-rays for 10 min. Finally, the fluorescence of the solution was measured to assess the generated ROS.

### Intracellular free radical production

The intracellular ROS production was investigated by the chemical probe 2, 7-Dichlorodi-hydrofluorescein diacetate (DCFH-DA), which could be oxygenated by the generated ROS and emits green fluorescence. The 4T1 cells were inserted into 12-well plates and grown with adhesion on the walls. The cells were further incubated for 6 h in the addition of fresh medium containing PtN_4_C-SAzyme (100 µg/mL). After washing three times with PBS, 4T1 cells were exposed to X-ray (6 Gy) for 5 min. Then, DCFH-DA was added and incubated with 4T1 cells for another 30 min. the production of ROS could be observed by CLSM.

### Measurement of cytotoxicity in PtN_4_C-SAzyme

The cytotoxicity of PtN_4_C-SAzyme was evaluated by the standard Cell Counting Kit-8 (CCK-8). The 4T1 cells were placed in 96-well plates at a density of 4 × 10^3^ cells per well and incubated in a humidified incubator (37 °C, 5% CO_2_) for 24 h. Each well was then washed with PBS (10 mM, pH = 7.4) and different concentrations of PtN_4_C-SAzyme (0, 6.25, 12.5, 25, 50, and 100 µg/mL) were added. The cells were then coincubated for 24 h. 10 µL of CCK-8 was subsequently added to each well and kept in the incubator for another 1 h. Finally, cell viability was assessed by absorbance at 450 nm using a microplate reader (Thermo Scientific, Multiscan MNK3). Cells without PtN_4_C-SAzyme were treated as controls, and cell viability was calculated relative to control cells.

### *In vitro* clonogenic survival assays and detection of DNA double-strand breaks

 To measure the sensitivity to DNA damaging agents, clonogenic survival assays were performed. The 4T1 cells were seeded at 1000 cells/well into 24-well plates and cultured for 24 hours. Then, the cells were divided into 4 groups including (Ⅰ) Control (without treatment), (Ⅱ) PtN_4_C-SAzyme, (Ⅲ) X-ray, (Ⅳ) PtN_4_C-SAzyme+X-ray (6 Gy). The cells after different treatments were further cultured for 10 days and then the colonies were stained with Giemsa dye and the survival rate of the colonies was used to assess the effect of the different treatments. To further test for DNA double-strand breaks, 4T1 cells were cultured at a density of 3 × 10^4^ cells per well in a 35 mm confocal plate. After 24 h, the cells were divided into four treatment groups: (Ⅰ) Control (without treatment), (Ⅱ) PtN_4_C-SAzyme, (Ⅲ) X-ray, (Ⅳ) PtN_4_C-SAzyme+X-ray (6 Gy). The Cells were fixed with 4 % paraformaldehyde (10 min) after 24 h of culture and permeabilized with 0.2 % Triton X-100 (10 min). Next, to prevent their interaction with nonspecific proteins, 4T1 cells were treated with 5% FBS for 1 h. The cells were previously incubated with anti-phospho-histone H2A.X (ser139) overnight at 4 °C, followed by Anti-rabbit IgG (H+L) for 1 h. Hoechst was used to stain the nuclei of the cells. DNA was shown in red and phospho-histone H3 (PH3) in green. Finally, the cells were imaged under a confocal laser scanning microscope after washing.

### *In vivo* antitumor efficiency evaluation

All the experimental protocols were approved by Sichuan Provincial Centers for Disease Prevention & Control. Mice bearing 4T1 breast tumors were randomly classified into five groups: (Ⅰ) Control (without treatment); (Ⅱ) X-ray; (Ⅲ) cisplatin; (Ⅳ) PtN_4_C-SAzyme; (Ⅴ) PtN_4_C-SAzyme+X-ray. Each group contained four mice respectively. When the tumor volume of mice reached 75 mm^3^, 20 μL of saline or PtN_4_C-SAzyme (2 mg/mL, 20 μL) were injected subcutaneously in the right leg of nude mice with 4T1 mammary carcinoma into the body for 24 h. It was notable that the concentration of platinum ions in the third and fourth groups was consistent. The mice were exposed to X-rays (6 Gy) for 5 min. The changes in body weight and tumor volume were recorded for 20 days during treatment. The tumor growth is measured by calculated as follows: V= W^2^×L/2, where L and W stand for the length and width of the tumor, respectively. The relative tumor volume was V/V_0_, where V indicates the tumor volume on various days and V_0_ represents the first day's tumor volume. Meanwhile, the body weight of mice was measured every other day. Finally, mice were sacrificed and tumors and major organ tissues from five groups were collected for hematoxylin and eosin (H&E) staining, and blood was collected from the fundus arteriosus of each group of mice for routine blood and blood biochemical tests. Animal studies were conducted in compliance with the guidelines of the Institutional Animal Care and Use Committee. The mice were discarded according to the standard approved protocol after we finished the experiment.

### Histological analysis

After treatment, mice were sacrificed and tumors and major organ tissues (heart, liver, spleen, lung, kidney) were collected from each group of mice, fixed in 4 % paraformaldehyde, and embedded in paraffin. 5 μm sections were taken, and the sections were examined histopathologically with hematoxylin and eosin (H&E). In addition, blood was collected from the fundus artery of each group of mice for routine blood and blood biochemical examination.

### Statistical analysis

All the experiments completed accomplished in triplicate. The data obtained were expressed as the mean value ± standard deviation, and the Student's t-test was performed to analyze the statistical significance between two groups. *p < 0.05 was considered statistically significant and **p < 0.01 means extremely significant.

## Supplementary Material

Supplementary figures.
