# Supplementary material for: Tumor microenvironment-activated single-atom platinum nanozyme with H2O2 self-supplement and O2-evolving for tumor-specific cascade catalysis chemodynamic and chemoradiotherapy
Source: Theranostics. 2022 Jul 4;12(11):5155–71. doi: 10.7150/thno.73039 (PMC9274735; doi:10.7150/thno.73039)
Supplement: Supplementary file 1 — Supplementary figures. [file thnov12p5155s1.pdf]

Supporting Information

Tumor microenvironment-activated single-atom platinum nanozyme with H_2O_2 self-supplement and O_2-evolving for tumor-specific cascade catalysis chemodynamic and chemoradiotherapy

Qiqi Xu[1,2#], Yuetong Zhang[1,2#], Zulu Yang[1,2], Guohui Jiang[6], Mingzhu Lv[1,2], Huan Wang[1,2], Chenghui Liu[1,2], Jiani Xie[4], Chengyan Wang[3], Kun Guo[5], Zhanjun Gu[3], and Yuan Yong[1, 2*]

1. Key Laboratory of Pollution Control Chemistry and Environmental Functional Materials for Qinghai-Tibet Plateau of the National Ethnic Affairs Commission, School of Chemistry and Environment, Southwest Minzu University, Chengdu 610041, China.

2. Key Laboratory of General Chemistry of the National Ethnic Affairs Commission, School of Chemistry and Environment, Southwest Minzu University, Chengdu 610041, China.

3. CAS Key Laboratory for Biomedical Effects of Nanomaterials and Nanosafety, Institute of High Energy Physics, Chinese Academy of Sciences, Beijing100040, China.

4. College of Pharmacy and Biological Engineering, Chengdu University, Chengdu, 610106, China.

5. College of Pharmacy, Southwest Minzu University, Chengdu 610041, China.

6. Department of Neurology, Affiliated Hospital of North Sichuan Medical College, Nanchong 637000, China.

[#] These authors contributed equally.

Corresponding Authors:

*E-mail: yongy1816@163.com

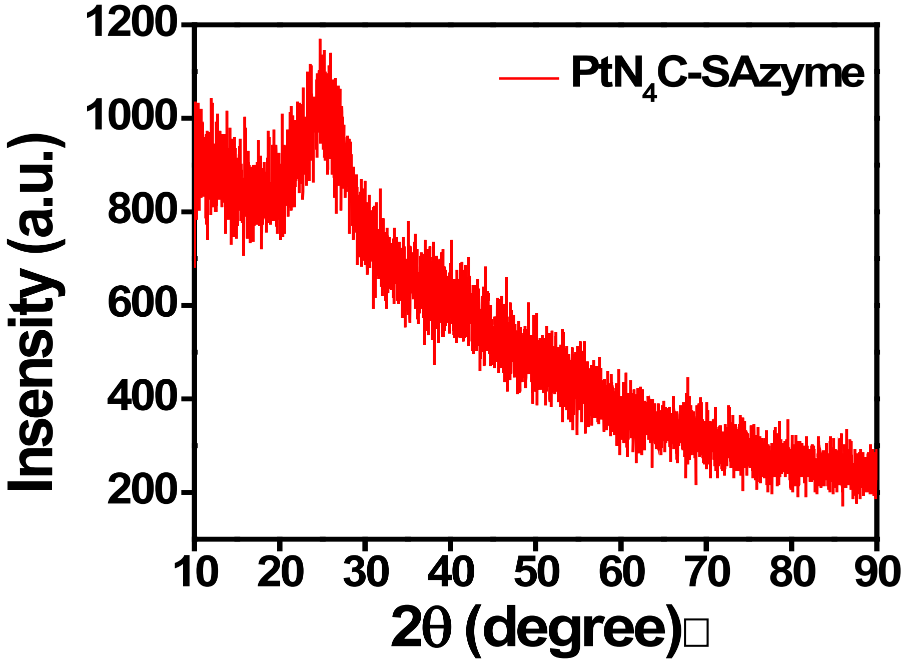

Figure S1. XRD patterns of PtN_4C-SAzyme, excluding the existence of Pt nanopartiles.

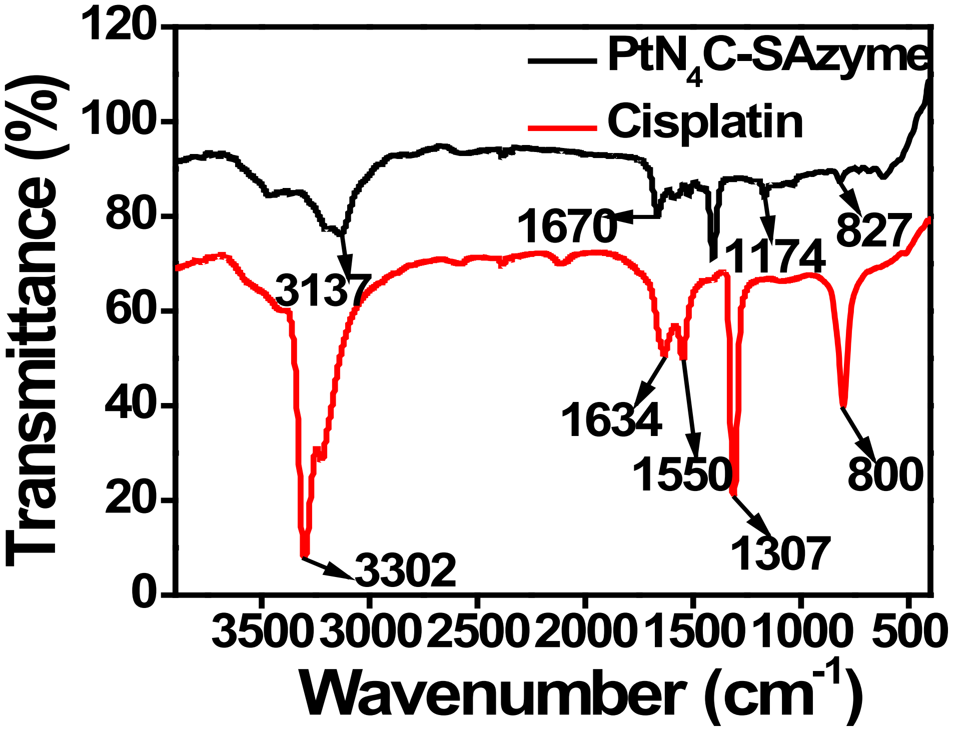

Figure S2. FT-IR spectrum of PtN_4C-SAzyme.

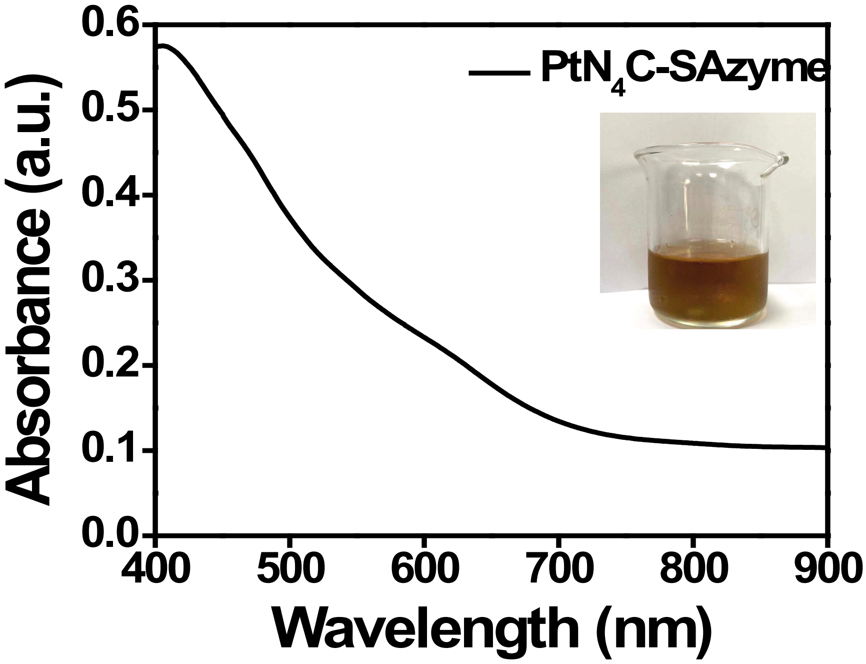

Figure S3. UV-vis absorption spectrum of PtN_4C-SAzyme.

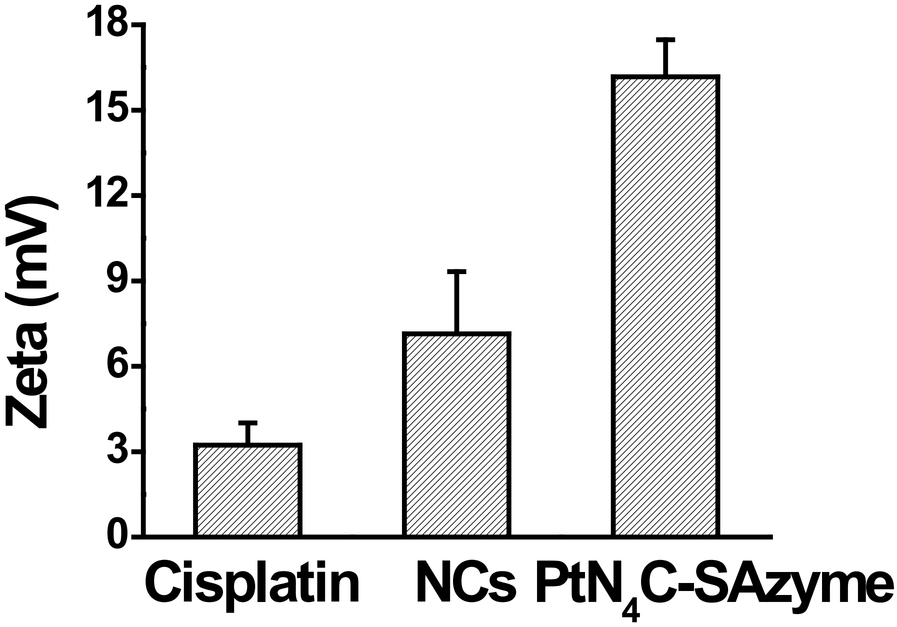

Figure S4. ζ potentials of cisplatin, NCs and PtN_4C-SAzyme.

Table S1. EXAFS fitting parameters at the Pt L3-edge for various samples

	Shell	N [a]	R (Å) [b]	σ^2 (Å^2·10^{-3}) [c]	ΔE_0 (eV) [d]	R factor (%)	Reference
Pt foil	Pt-Pt	6	3.91	5.58	7.41	1.3	Nano Energy., **2020**, 74, 104931
PtO_2	Pt-O	6	1.98	0.73	7.05	3.5	
PtN_4C-SAzyme	Pt-N	4	2.01	6.2	12.1	0.9	This work

[a] *N*: coordination numbers; [b] *R*: bond distance; [c] σ^2: Debye-Waller factors; [d] ΔE_0: the inner potential correction. *R* factor: goodness of fit. S_0^2 was set as 0.86 for Pt-N, which was obtained from the experimental EXAFS fit of reference PtO_2 by fixing CN as the known crystallographic value and was fixed to all the samples.

Error bounds that characterize the structural parameters acquired by EXAFS spectroscopy was evaluated as N ± 20%; σ2 ± 20%. The accuracy range was estimated as ΔE_0 ± 0.03A.

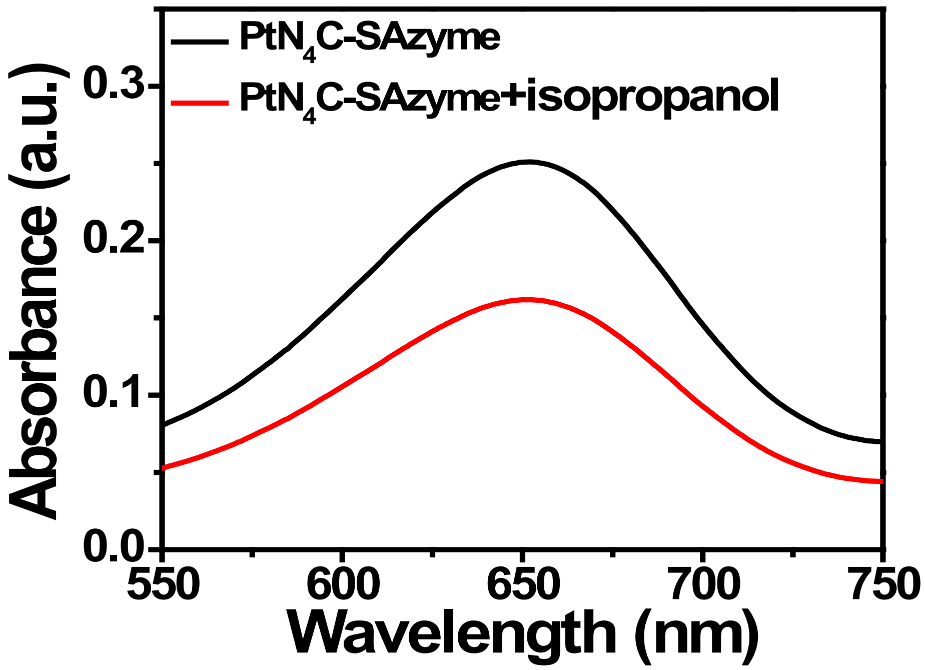

Figure S5. UV-vis absorption spectrum of PtN_4C-SAzyme-H_2O_2-TMB system in the presence or absence of isopropanol.

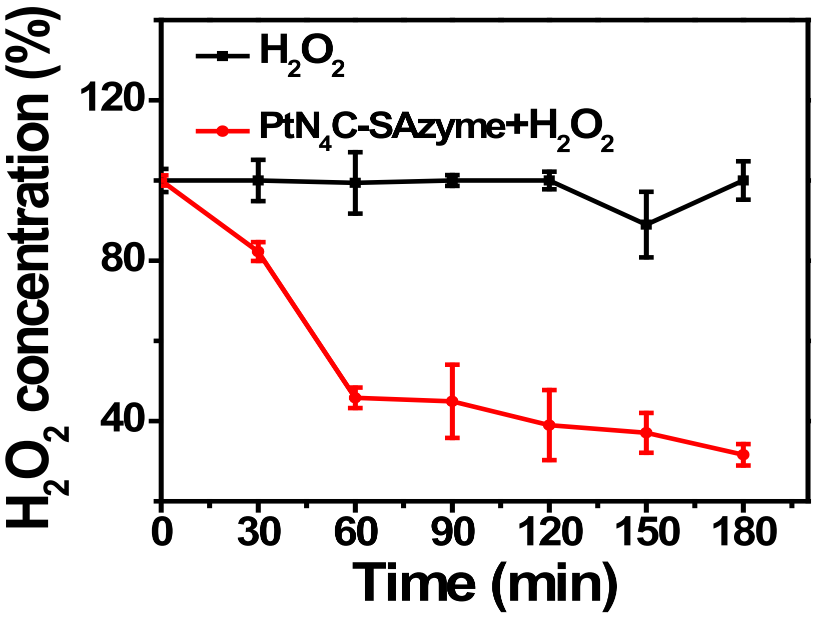

Figure S6. Concentration of H_2O_2 before and after being incubated PtN_4C-SAzyme aqueous solution with different time.

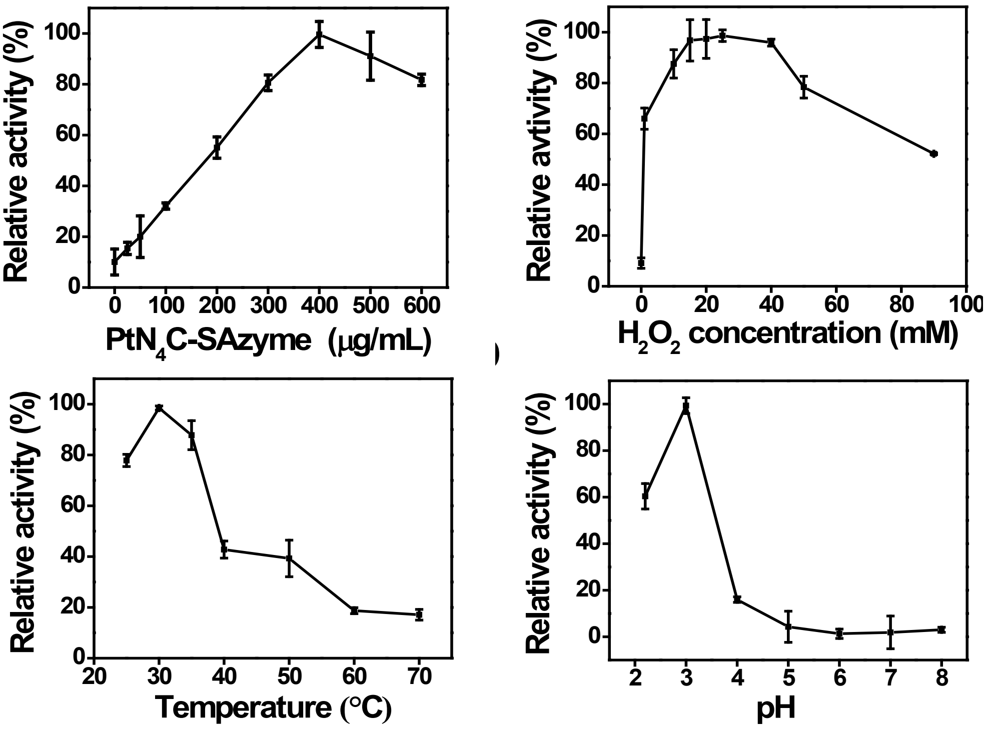

Figure S7. The peroxidase-like activity of the PtN_4C-SAzyme is sample (A) and H_2O_2 concentration (B), temperature (C), and pH (D).

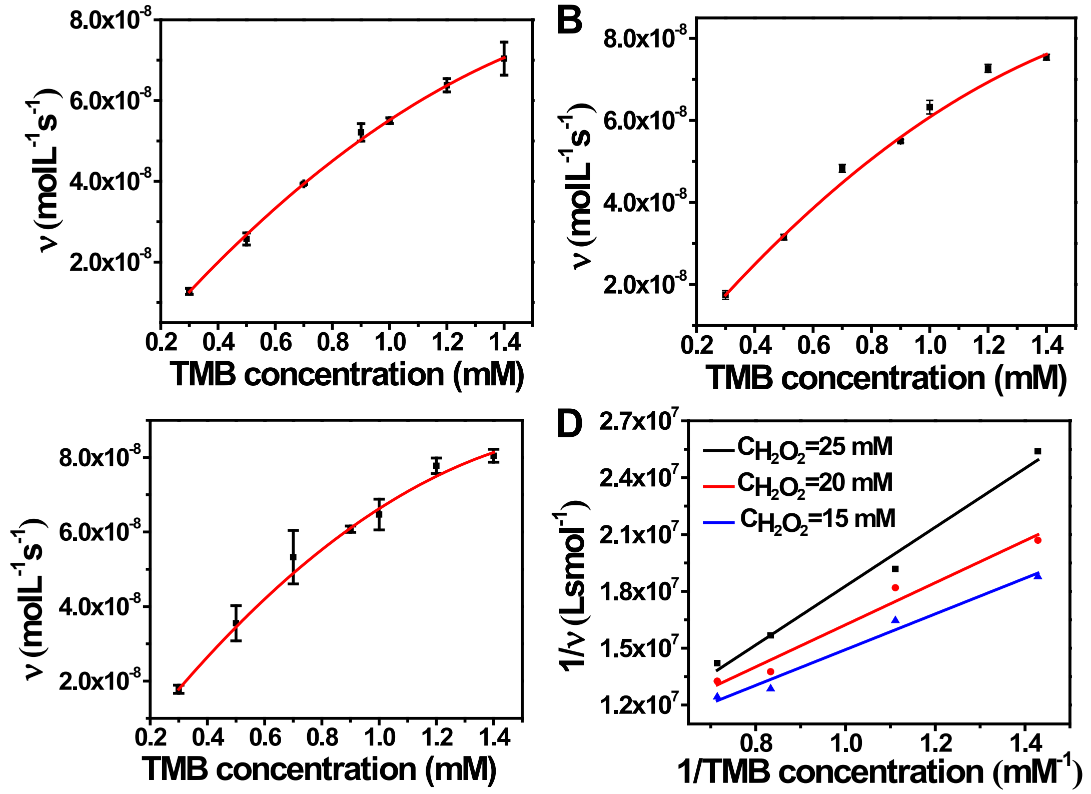

Figure S8. (A-C) The velocity (v) of the reaction was measured using 100 µg/mL of PtN$_4$C-SAzyme in different concentration of H_2O_2 as a function of TMB. (D) Double-reciprocal plots of activity of PtN$_4$C-SAzyme at a fixed concentration of TMB versus varying concentration of H_2O_2.

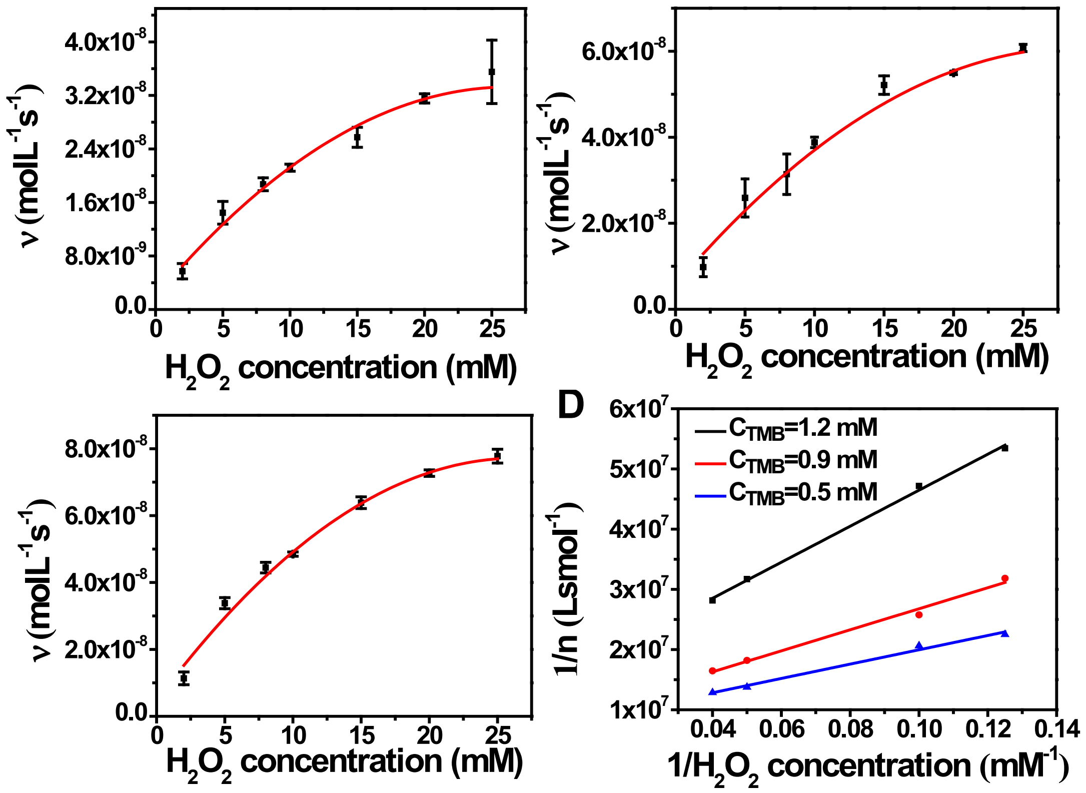

Figure S9. (A-C)The velocity (v) of the reaction was measured using 100 μg/mL of PtN$_4$C-SAzyme in different concentration of TMB as a function of H_2O_2. (D) Double-reciprocal plots of activity of PtN$_4$C-SAzyme at a fixed concentration of H_2O_2 versus varying concentration of TMB.

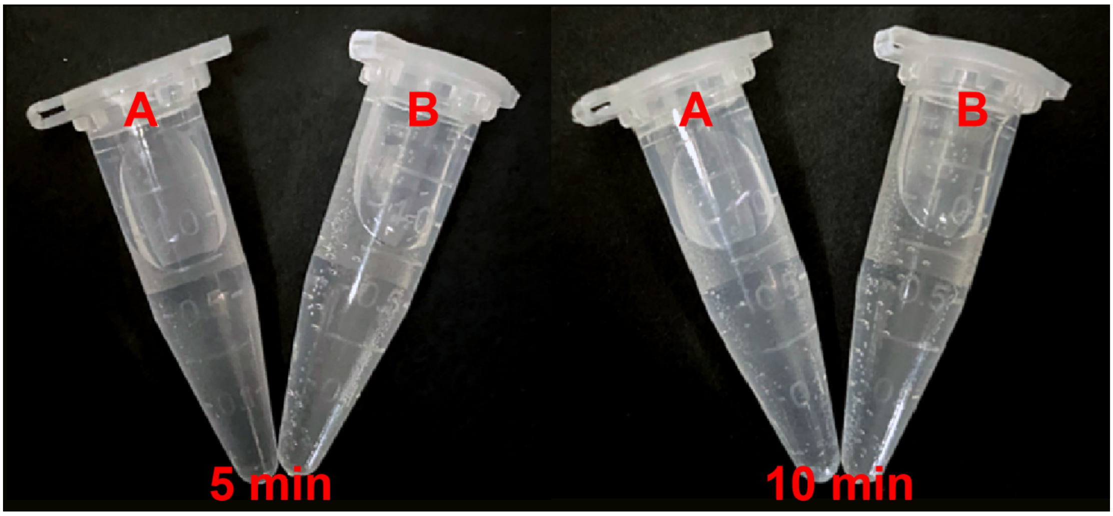

Figure S10. The content of dissolved oxygen before (A) and after (B) being incubated with PtN_4C-SAzyme aqueous solution with different time.

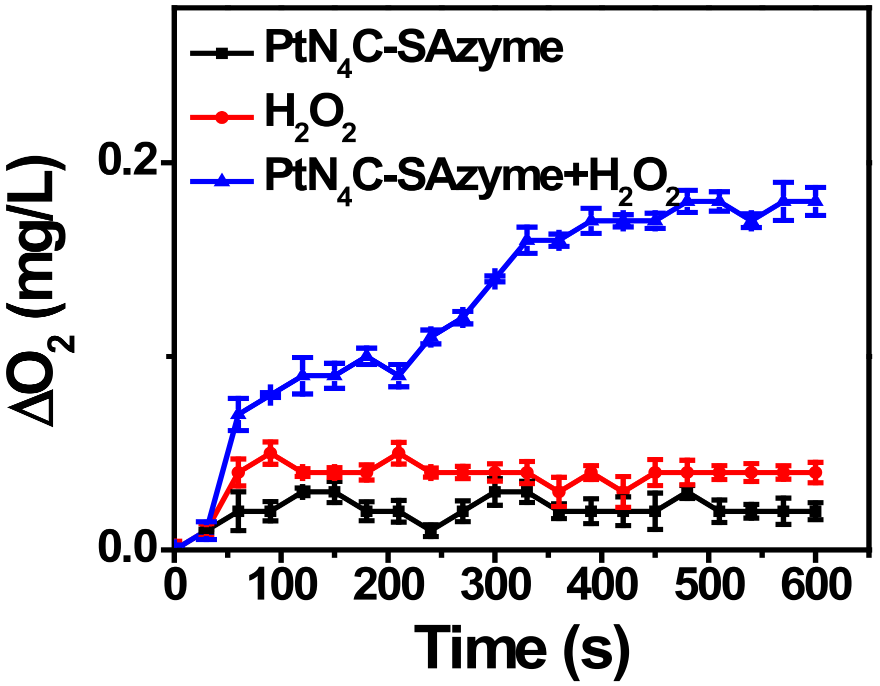

Figure S11. O_2 generation in different systems in the absence or presence of PtN_4C-Sazyme or H_2O_2 at pH 5.0.

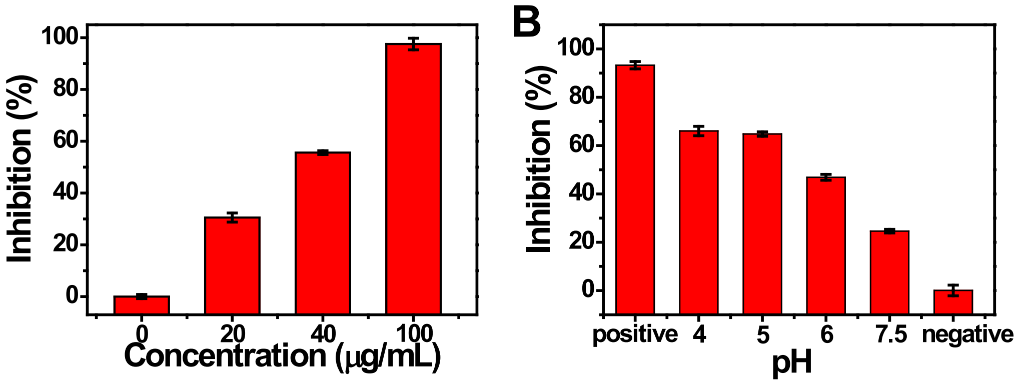

Figure S12. The inhibition of $O_2^{\cdot -}$ by the disproportionation of PtN_4C-SAzyme under different concentration and pH.

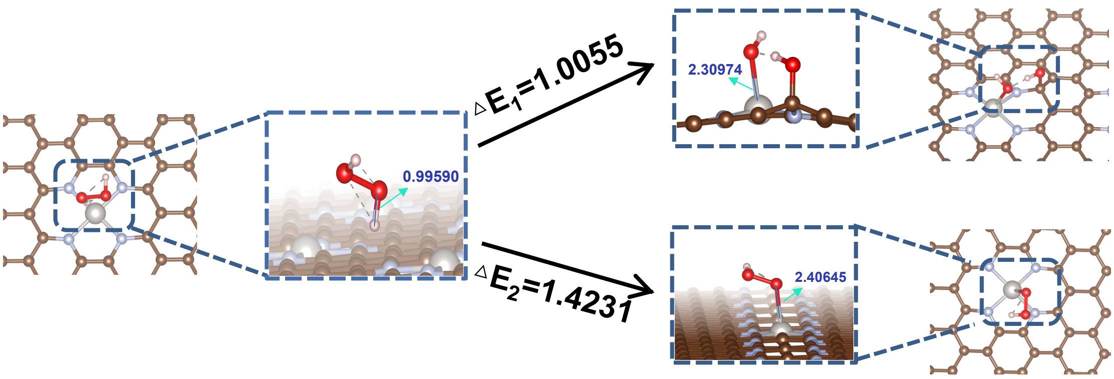

Figure S13. A reaction process of decomposing H_2O_2 into produce 2OH* and O*_H_2O* by hemolytic and heterolytic path, respectively.

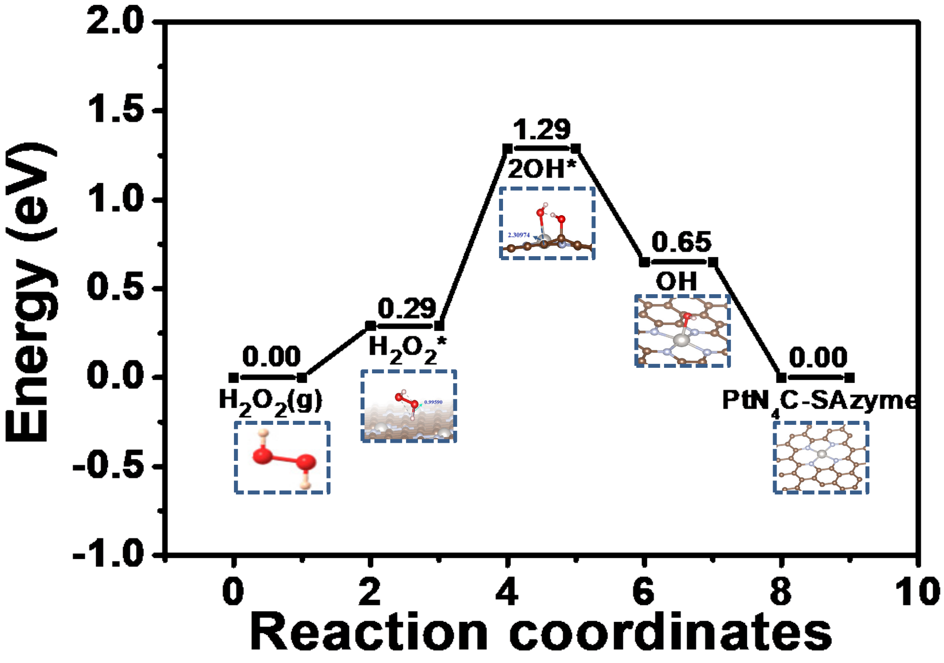

Figure S14. Free energy diagram of PtN_4C-SAzyme for decomposing H_2O_2 into produce 2OH* by hemolytic path.

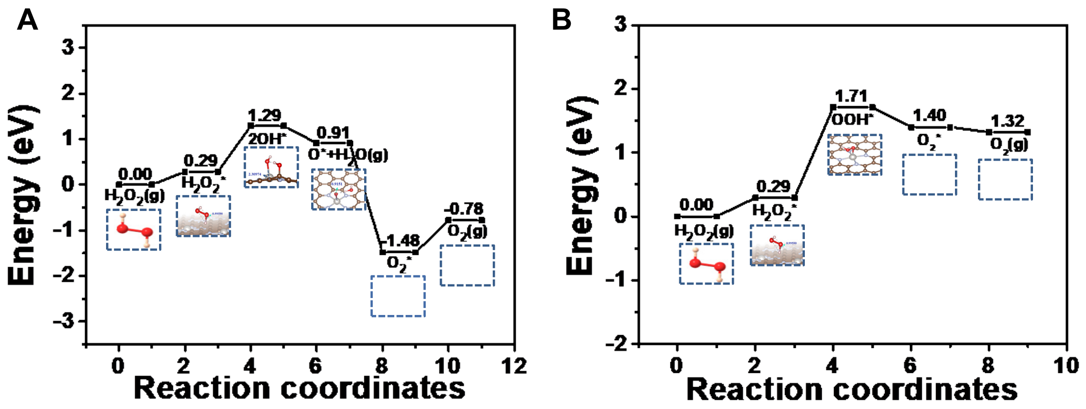

Figure S15. Free energy diagram of PtN_4C-SAzyme for catalytic oxidation of H_2O_2 into O_2 by generating the intermediates of produce O* + H_2O and OOH*, respectively.

Figure S16. The level of GSH incubated with different concentration of PtN_4C-SAzyme (A) and different time (B).

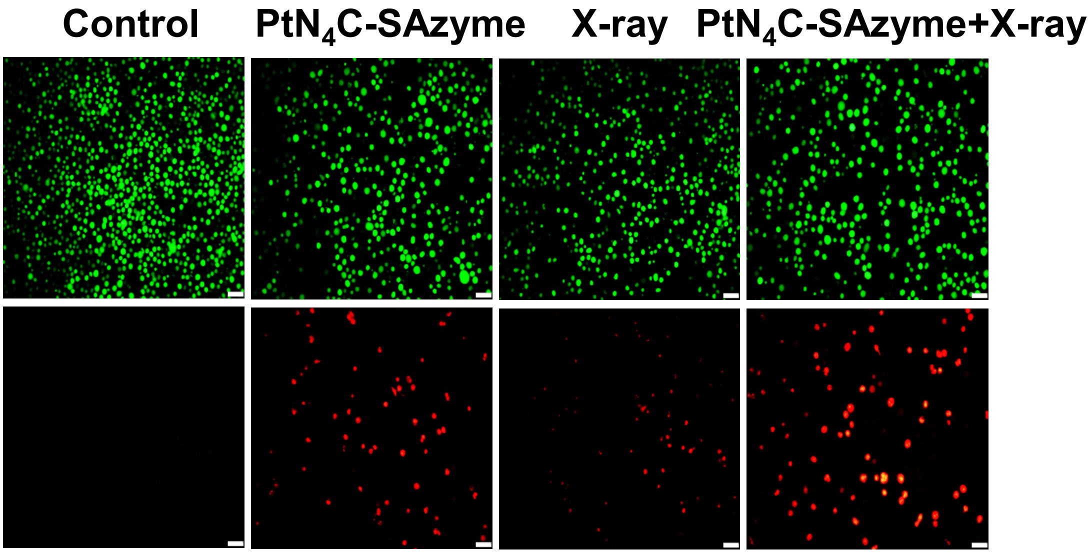

Figure S17. Live/dead co-staining images of 4T1 cells in different solutions. Scale bar=20 μm.

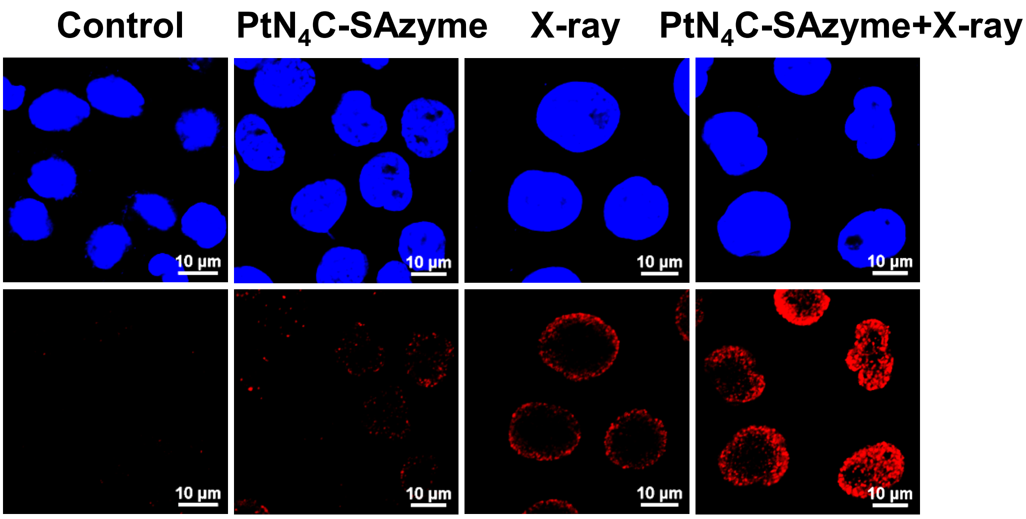

Figure S18. Representative nuclear condensation and DNA fragmentation induced by PtN_4C-SAzyme (10 μg/mL, 2 mL) and/or X-ray radiation (6 Gy).

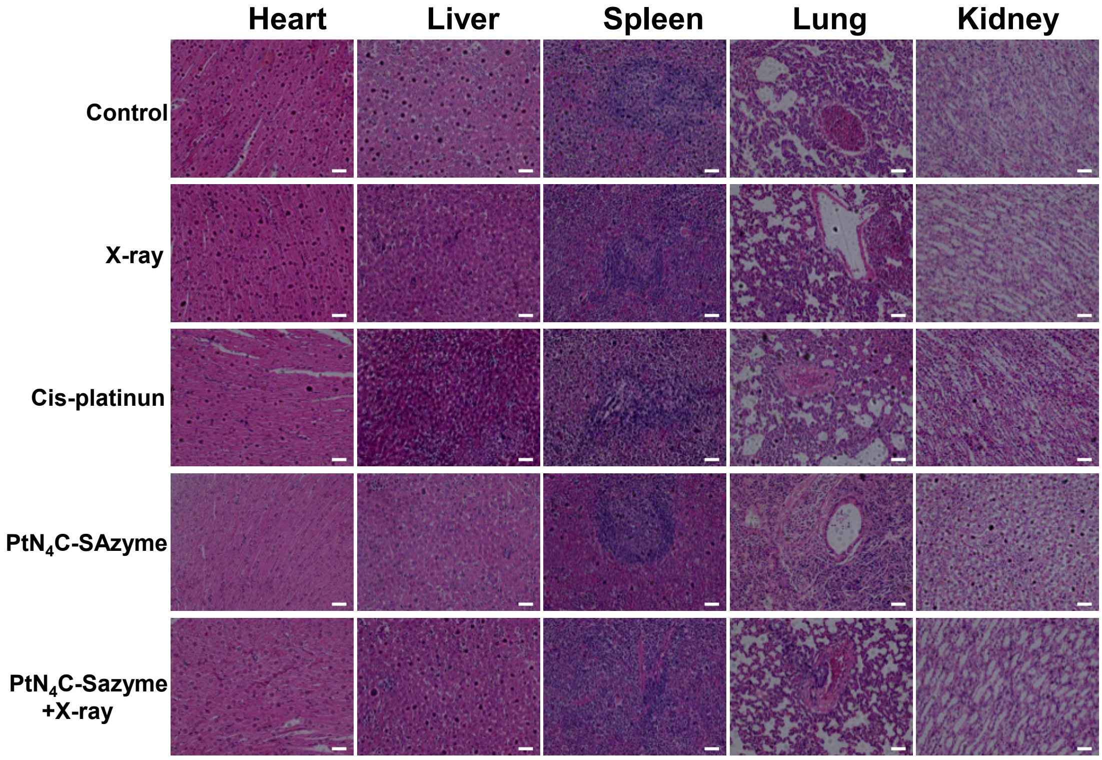

Figure S19. H&E staining assay of main organs containing heart, liver, spleen, lung and kidney in different groups. Scale bar=50 μm.

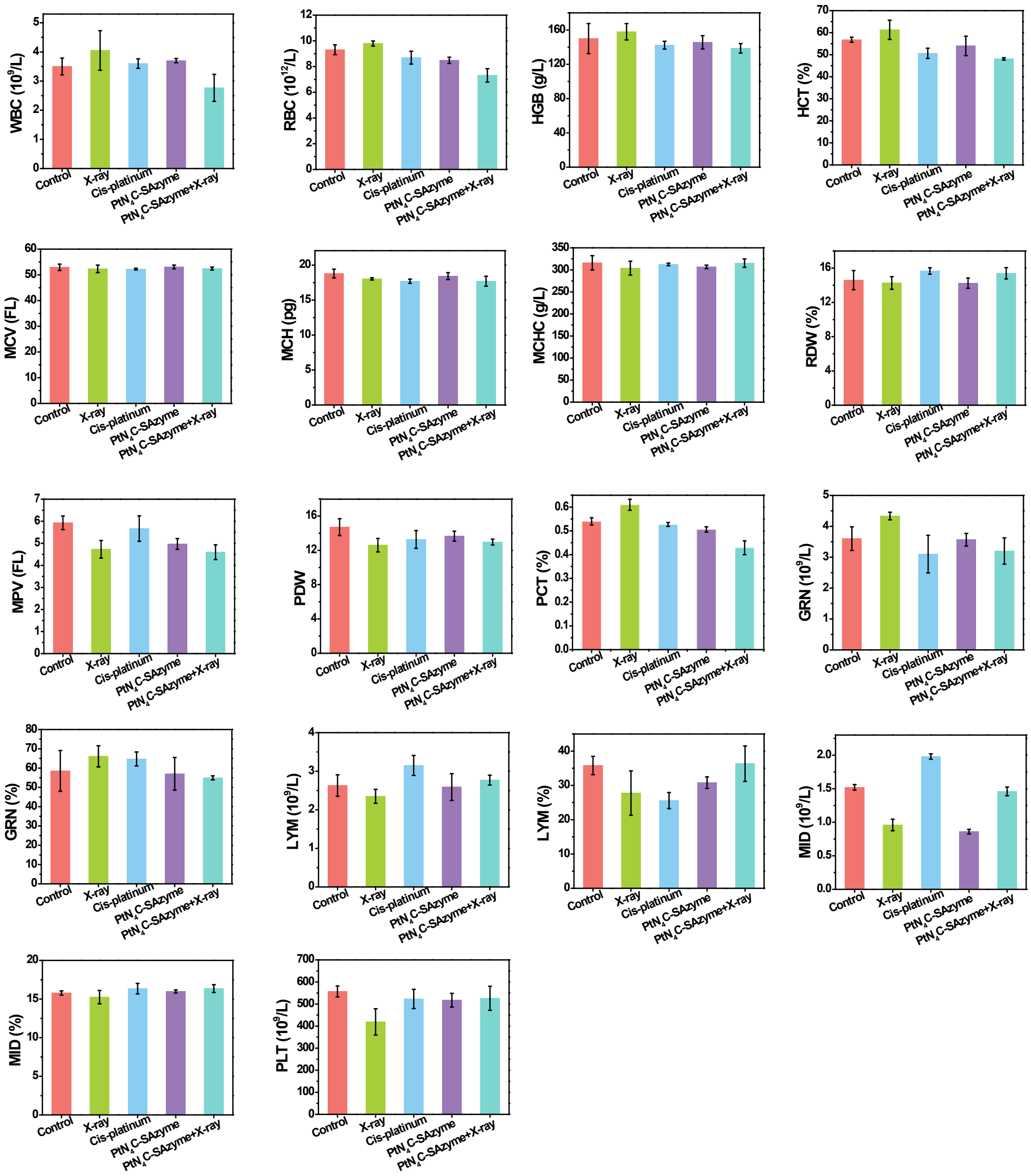

Figure S20. Hematology analysis of mice in different treatment.

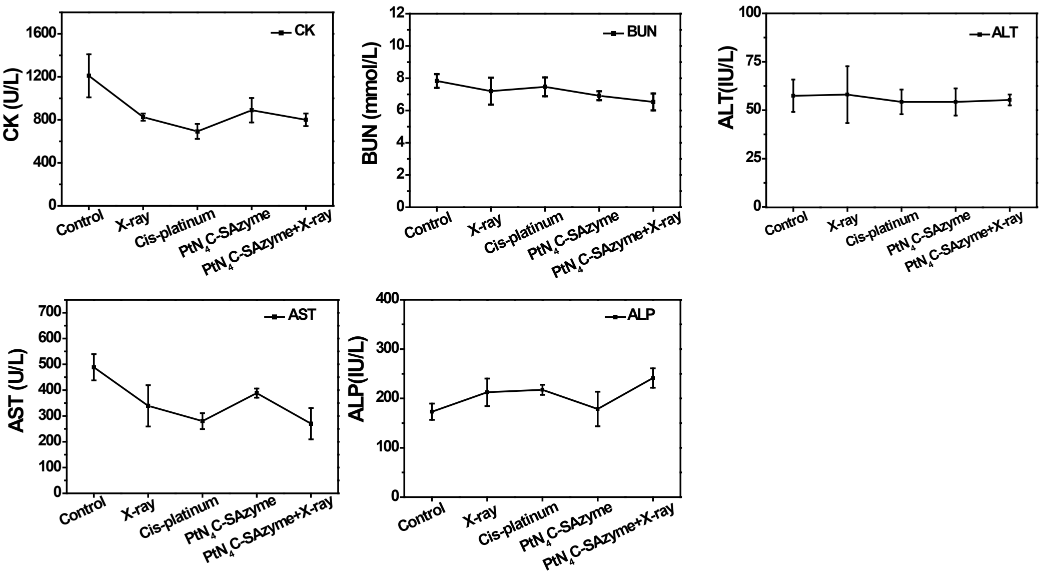

Figure S21. Blood biochemistry analysis of mice in different treatment.
